# Extreme social isolation risk is associated with story-driven, strategic and cooperative-first gameplay preferences

**DOI:** 10.1371/journal.pmen.0000517

**Published:** 2026-07-29

**Authors:** Brian Confessor, Monica Perusquía-Hernández, Kongmeng Liew, Panote Siriaraya, Yoriko Matsuda, Erika Kimura, Yutaro Hirao, Hideaki Uchiyama, Kiyoshi Kiyokawa

**Affiliations:** 1 Nara Institute of Science and Technology, Ikoma, Japan; 2 University of Canterbury, Christchurch, New Zealand; 3 Kyoto Institute of Technology, Kyoto, Japan; Santa Casa de Sao Paulo School of Medical Sciences, BRAZIL

## Abstract

Video-games could help players deal with *hikikomori* – extreme social withdrawal. However, it is still unclear what game elements are preferred by this audience, and thus it is difficult to design game-based interventions for them. To address this gap, we investigated the gaming preferences of hikikomori players through a survey (*N* = 529). To focus on those at higher risk of becoming hikikomori, participants were categorized into High and Low Hikikomori Risk (H-NHR and L-NHR) using the NEET-Hikikomori Risk (NHR) scale. Open-ended questions were asked about participants’ opinions towards favourite games, single-player, multiplayer, and online games. We quantitatively compared favourite game genres and hikikomori risk tendencies, finding no significant relationship between the two groups. Inductive thematic analysis showed that H-NHR players reported experiencing more negative player interactions, preferring cooperative over competitive gameplay. These resonate with the heightened social anxiety and sensitivity to social rejection that have been known to exacerbate the hikikomori condition. Moreover, there were several positive mentions of action, adventure, narrative, and strategic gameplay elements from the H-NHR group. From our findings, we summarise game design features that could be used in future game-based interventions to help hikikomori players overcome their social withdrawal without worsening their conditions, such as short play sessions, simple mechanics, involving narratives and location-based elements.

## Introduction

*Hikikomori* refers to a severe form of social withdrawal that has affected millions of people worldwide over the last decades, and is particularly pronounced in Japan [1]. The hikikomori phenomenon was first popularised by Tamaki Saito in the 1980s, and hikikomori individuals were characterised by an extreme withdraw from social activities and interactions for a prolonged period of time [2]. Koyama and colleagues [3] estimated Japanese prevalence rates at 1.2% of individuals between 20 and 49 years of age, who have experienced “hikikomori” at some point in their lifetime. These skewed towards male youth and young adults, and 54.5% of them also reported some psychiatric disorder in their lifetime, such as depression or anxiety.

Although the phenomenon is well-studied and documented in Japan, it is no longer considered a Japanese culture-bound syndrome. Instead, the hikikomori condition seems widely present and studied in many parts of the world [4], such as Italy [5], Brazil [6], the Middle East [7], Malaysia [8] and the United States [9]. The social isolation that accompanies hikikomori can give rise to negative effects on the affected people and those around them, such as friends and family [10,11]. These can be exacerbated by comorbities such as depression, further affecting the individual’s mental health and self-perception. Other comorbidities, such as Autism Spectrum Disorder (ASD), have also been perceived in hikikomori participants. In a pilot case–control study from Katsuki et al. [12], hikikomori participants were more likely to have higher autistic tendencies, based on the Autism Spectrum Quotient [13] questionnaire. Similarly, Brosnan & Gavin [14] have shown that higher levels of autistic traits could serve as a mediator to increased hikikomori risk in young adults. Given these overlaps, it is important to consider neurodiversity when studying hikikomori, as depressive and autistic traits may influence not only their withdrawal levels, but also the way hikikomori people may engage with leisure and social activities, such as video games. These findings suggest links between the hikikomori and ASD, also making it more difficult to understand the limits and properly characterize the hikikomori phenomenon and its separation from other previously established conditions.

In investigating the hikikomori phenomenon, Uchida and Norasakkunkit [15] identified a set of underlying psychological tendencies stemming from cultural marginalisation, such as low confidence in one’s abilities and a preference for casual or part-time work, that were strongly predictive of Hikikomori behaviour. This categorises the hikikomori condition not just as a behavioural or sociological phenomenon, but also as a psychological condition. As with other internalising psychopathologies like depression and anxiety, hikikomori *risk tendencies* can be identified not only by behavioural markers of social withdrawal, but also motivational and attitudinal profiles: the extent to which individuals *would like* to withdraw from society. As such, in our paper, we adopt this definition of hikikomori as a *psychological* condition, rather than a behavioural condition.

Because of the problems associated with hikikomori tendencies, approaches have been sought to help those afflicted. When physically and socially isolated, hikikomori individuals could turn to video games as a common pastime, or source of social interaction. On the one hand, this can be problematic since video games may be addictive, and problematic gaming behaviours (such as internet gaming disorders) are often linked to hikikomori conditions [7,16,17]. On the other hand, video games are a promising medium that may lead to behavioural change and well-being management when well designed. A systematic review on the effects of active video games on mental health among college students showed that active video games could improve stress, anxiety and depression of college students, as well as increasing their happiness and psychological satisfaction [18]. Past research has also shown how games can improve mental health in specific cases, such as moments of difficulty [19], in coping with grief and loss [20], and in potentially managing symptoms of General Anxiety Disorder [21]. Past research from Trepte, Reinecke & Juechems [22] has also shown that online social capital acquisition in gaming can result in strong social ties, and may also predict offline social support if the game activities are extended to the offline world. Similarly, Martončik & Lokša [23] found that World of Warcraft players reported experiencing less loneliness and social anxiety in the game, compared to the real world. This could mean that games with social features could offer simpler and safer socialization options for those who are more lonely or socially anxious, offering a “practice space” for social capital bridging before going into the offline world.

Furthermore, this potential in behavioural change has been backed up by some psychological frameworks such as the Self-Determination Theory (SDT) [24]. The SDT claims that people can better maintain their emotional well-being when three intrinsic psychological needs (Autonomy, Competence and Relatedness) are met. Past research has shown that, through careful game design, video games are able to fulfill all three of these intrinsic needs [25,26]. Therefore, well-designed games could potentially foster motivation and emotional regulation in hikikomori players.The potential of game-based interventions for reaching socially isolated audiences has been demonstrated by Chan [27] and Khiatani et al. [28], while Aguglia et al. [29] suggests that Virtual Reality can be an approach to target hikikomori individuals specifically. These findings reinforce the potential of game-based interventions as a first step towards behavioural change, which could potentially improve socialization over time alongside the use of traditional therapy alternatives.

In order to aid hikikomori people to go out more and potentially socialize, Location-Based Games are often cited as an interesting genre to use towards this goal [30,31]. This is because these types of games incorporate leaving one’s home as part of their gameplay, by gamifying the rehabilitation process back towards societal reintegration. For instance, several studies such as Evans et al. [32] and Finco [33] discuss how Pokémon GO, one of the biggest Location-Based Games available, can lead players towards social interactions and making new friends, which in turn leads to an increased social playing time and increased positive perceptions of the game.

Considering that games could be a double-edged sword that could lead to other problems, such as Internet Gaming Disorder [7,16,17], it is crucial to understand how the features present in games can improve hikikomori conditions and not exacerbate them. To this end, we believe the answer lies in understanding how and why hikikomori individuals play video games. To our knowledge, our research is the first to investigate what game elements and genres are preferred by those at high risk of becoming hikikomori, and how their play styles might differ from neurotypical players. Through this knowledge, we aim to provide a foundation for other research to develop game-related therapeutic approaches for this audience. If appropriately designed, games could help support hikikomori individuals psychologically, such as helping increase their sense of self-worth, and even facilitate their rehabilitation towards a more social lifestyle.

As such, the present study intends to investigate Japanese hikikomori players’ gaming preferences by conducting a survey with participants with a high risk of becoming hikikomori, using the NEET-Hikikomori Risk (NHR) Scale [15] to measure participants’ risk of social isolation. From their opinions, we will investigate and extract game design suggestions, categorizing them based on which of the three intrinsic needs of the SDT may be fulfilled by each design suggestion (Autonomy, Competence or Relatedness). Given that our goal is to suggest game design that can lead to behavioural change, we utilized the SDT as a way to categorize the game design suggestions extracted from the results. This way, it is possible to design games that may lead to a positive social change in player behaviour, as seen in past research related to the SDT frameworks [25,26]. The survey’s results can provide a better understanding of hikikomori player preferences and game design suggestions for hikikomori players. With this knowledge, game developers and researchers who wish to create game-related interventions for hikikomori audiences would better know game elements favoured or disfavoured by these players. This could, in turn, increase player engagement and, subsequently, the chance of a successful intervention. Given that this paper focus mainly on hikikomori risk levels, and some hikikomori individuals may also present autistic traits [12,14], differences in communication preferences or styles of interaction could also change the way that games are approached by these audiences. We thus frame our findings with this caveat in mind.

Therefore, this paper aims to answer the following questions:

**RQ1 - Is there a relationship between a person’s NHR level and their gaming preferences?**
**RQ2 - How does a person’s NHR level relate to opinions about playing with other people?**
**RQ3 - Which game genres and features are effective in engaging with players with high NHR levels?**

To address RQ1 and RQ2, we developed a survey to learn more about the video game preferences of people with varying degrees of NHR levels, and conducted a mixed-method analysis of their opinions on the topic. This mixed-methods analysis was comprised of a quantitative analysis using Chi-Square tests to investigate possible relationships between hikikomori risks and game genres, while the qualitative analysis was comprised of inductive thematic analysis on players’ opinions on certain kinds of games. From the understanding of RQ1 and RQ2, we provide game feature requirements to answer RQ3.

The main contributions of this paper are:

- Understanding the nuances of differences between high and low-risk hikikomori players on their social gaming preferences. For instance, understanding the preferences of both groups about cooperative and competitive gameplay.
- Understanding gaming preferences of high-risk hikikomori players on various game elements, such as world building, narrative, puzzle, action, and role-playing mechanics.
- Summarising a list of game design suggestions, grounded on the Self-Determination Theory’s intrinsic needs for well-being management, that could be used for video games targeted to an audience with high risk of becoming hikikomori, both for commercial games and game-based interventions.

## Related works

### Hikikomori and hikikomori risk tendencies

Aside from the popularised definition given by Saito [2], other researchers have given similar definitions for the condition. For instance, Teo & Gaw [1] understand hikikomori as a possible culture-bound syndrome that may or may not meet acceptance criteria as a new psychiatric disorder. For Teo & Gaw [1], hikikomori could be more related to social elements in the Japanese culture than an actual new condition. Kato et al. [34], however, define that a person suffering from hikikomori must be socially isolated in their home for at least six months and suffer significant functional impairment or distress associated with such isolation. They also sub-categorise hikikomori into mild, moderate, or severe, depending on the duration of their isolation.

Our research follows the cultural marginalisation theory of hikikomori, where much like NEET (Not in Employment, Education or Training [35]) individuals, posits that it is a continuous spectrum rather than a binary diagnosis that arises from individuals failing to fit in with their cultural environment. To measure one’s risk tendencies of becoming hikikomori, the NEET-Hikikomori Risk (NHR) Scale [15] was developed. This scale considers the hikikomori and NEET conditions not as two separate diagnoses, but rather on a “spectrum of psychological tendencies associated with the risk of being marginalized in society,” by considering commonalities in psychological tendencies between the two conditions. This scale has also been shown to correspond strongly to other behavioural and cognitive measures in hikikomori [36].

In the NHR Scale, three main factors are used to gauge the psychological tendencies behind hikikomori risk: (1) “Freeter” lifestyle preference, which, in the Japanese context, refers to the tendency to consciously choose to not work despite having possible job availabilities, (2) a lack of self-competence, and (3) unclear ambitions for the future. During the COVID-19 pandemic, society was forced to adopt more isolated lifestyles indoors. Post-pandemic, with the growing number of remote home office job positions, many chose to continue this lifestyle, which clashed with some earlier definitions of hikikomori, as people became more isolated but did not necessarily suffer many negative effects because of it. With this in mind, Kato, Sartorius & Shinfuku [37] found it important to distinguish between pathological and non-pathological hikikomori. They argue that, while physical isolation itself is not pathological, it can lead to mental disorders triggered by social isolation if these isolated people are not happy with their isolation. If NEET-Hikikomori exists on a spectrum, poor mental health may exacerbate non-pathological hikikomori conditions and lead to a downward spiral towards pathological states. Moreover, some hikikomori still suffer financial and mental difficulties over this isolation. Therefore, aiding these individuals before their conditions worsen is an important task for governmental, medical and academic entities that study this phenomenon, and using video games with grounded design choices is one approach towards this goal.

### Self-Determination Theory (SDT) and the motivational pull of games

We posit that video games may be used as mechanisms to maintain or improve the mental health of hikikomori individuals, as they have been shown in past research to be effective in behavioural change and management of well-being. One of the well-grounded psychological foundations used to connect game mechanics to players’ emotional improvement is the Self-Determination Theory (SDT). The SDT [38] is a psychological framework that aims to explain human motivation based on three intrinsic psychological needs that must be fulfilled:

- **Autonomy:** The freedom over one’s choices, and the need to feel in control of one’s own actions and decisions;
- **Competence:** The need to feel capable and skilful when interacting with one’s surroundings, and to experience growth and mastery of skills;
- **Relatedness:** The need to feed connected, cared for, and part of a social group, and to have meaningful social interactions.

According to the SDT, by fulfilling these three intrinsic needs, people can feel more intrinsically motivated, interested in engaging in activities, and generally experiencing greater well-being. On the other hand, if those needs are not met, people may present impaired psychological health and lower motivation.

Video games have been shown to support these needs through carefully designed mechanics [25,26]. For instance, challenge-based mechanics and progression systems like those commonly found in Role-Playing Games could fuel player’s need for Competence; open-world exploration, non-linear gameplay and branching job systems that allow for greater character customization may aid in their Autonomy needs; and player interactions with other players, or even with non-playable characters in inside game narratives, could foster feelings of connection, which can help with their Relatedness needs.

SDT provides a structured way to understand emotional engagement and motivation, and thus it can serve as a useful framework to understand how good game design can positively impact players’ well-being. Past research showed that satisfying such needs can lead to an increase in play time [39] and player enjoyment [40]. Given these potential benefits, structuring our game design recommendations based on SDT allows game designers to intentionally develop mechanics that may fulfill these three basic needs, potentially making games more effective as intervention tools.

### Video games as interventions

Several previous studies have used video games as therapeutic interventions to improve some aspect of their players mental health and overall well-being, by making players go outside and exercise more often, or helping them deal with difficult emotions and situations.

One game genre that has shown extensive use in taking people outside is the location-based genre. Santos et al. [41] used location-based games to encourage senior citizens in a Japanese city to exercise more often, by developing a game that makes players go to landmarks around Kyoto. This resulted in players going out and exercising more often than before the intervention. Similarly, Vella et al. [42] conducted interviews on participants that played Pokémon GO to determine the possible social outcomes of playing, and what game mechanisms could foster social connection through engagement in the real world. From these interviews, thematic analysis was done on the participants’ answers. One theme that that stood out was “shared passion”, which was described as a desire to share the game with friends and family, becoming a key motivation to play the game. This shared passion was most evident in long-term fans of the Pokémon franchise, and being able to share this passion with others facilitated player connections, which could, in turn, strengthen social ties. Furthermore, these social interactions happened when players were encouraged to play outside, due to the geo-mapping features of Pokémon GO. These social consequences were further facilitated by the integration of game playing into players’ everyday lives.

Laato et al. [43] also conducted a field experiment using the Routes feature in Pokémon GO, finding that using this feature drove people outside more often, particularly when they offered more in-game rewards. This increased interest in playing could be understood as an operant conditioning [44], where the consistent positive reinforcement of receiving items as one goes through a Route could strengthen the association between location-based exploration and reward, thus increasing the frequency of the desired behavior. In this case, outdoor activity and exercise were the desired behaviours. The usage of Routes closer to population concentrations was also more popular, though participants in the study did express wishes to have Routes in rural and nature areas. These preferences towards urban areas and areas where players lived or spent their daily lives were corroborated in research by Colley et al. [45].

Aside from physical benefits, past research investigated how serious games and different game elements could help players’ mental well-being. For instance, Mirhadi et al. [19] illustrated how games could be used to help players during difficult moments in their lives, investigating the different game element that could be used as coping mechanisms. Such elements included challenges that could be completed, leading to a sense of control and accomplishment, or multiplayer modes and online communities that could provide emotional support to players in times of need.

Similarly, Catalan et al. [21] investigated how the combination of walking simulators and storytelling could help players deal with Generalized Anxiety Disorder (GAD), analyzing certain game elements that could help dealing with this condition. Elements like the absence of combat or time pressure, using “safe spaces” to gradually expose players to anxiety-inducing scenarios, the inclusion of virtual companions for an added sense of security, and environmental storytelling that makes players reflect on certain topics could all play a part in helping players build tolerance towards uncertain situations and potentially help them in dealing with their anxious feelings. Their findings also provided preliminary suggestions of how safe spaces in walking simulators could be reshaped to be used as a form of Graded Exposure Therapy, which could in time benefit players with their anxiety condition.

Researchers also investigated how games such as Spiritfarer could be used by players to help them cope with feelings of grief and loss [20,46].They discovered that gamers’ perspectives on the grieving and bereavement process were influenced by the design principles of the game related to emotional reactions and relationships between the story, characters, and players. These principles helped gamers manage their loss and sometimes reevaluate it.

### Hikikomori and video games

A challenge of using video games as a therapeutic intervention for hikikomori is the association between hikikomori and problematic gaming. A cross-sectional study done by Stavropoulos and colleagues on American and Australian hikikomori populations showed a positive correlation between hikikomori symptoms and higher risks of Internet Gaming Disorder, which is a condition listed on the Diagnostic and Statistical Manual for Mental Disorders (DSM-5), characterised by *“a persistent and recurrent use of the internet to engage in games (8-10h of gameplay per day, and at least 30h per week), often with other players, leading to clinically significant impairment or distress”*. Similarly, Kato and colleagues found increased internet usage and gaming addiction in Japanese hikikomori [47], while Kubo et al. [17] found that pathological hikikomori, which are defined by those who are physically isolated and also have significant distress and/or various functional impairments [48], have shown higher scores in gaming disorder tendencies. These findings show the darker side of what the misuse and over-usage of games can cause.

While this suggests that games may be harmful for hikikomori individuals, it also suggests that hikikomori individuals may actually be receptive to game-based interventions, given its strong link with persistent and recurrent gaming. Therefore, there is still hope for using this media for hikikomori audiences to bring about positive changes.

Games have been extensively used in research with positive results in areas such as mental health therapy and cognitive rehabilitation. Some game users described that the games helped as coping mechanisms in difficult times [19], or to deal with and go through moments of grief and loss [20,46]. There is also preliminary evidence that games could be helpful in managing General Anxiety Disorder [21]. Because of these promising findings, it could be the case that newer media such as virtual reality and video games could also be effective tools in connecting to socially isolated players.

For example, Aguglia et al. [29] suggests that VR interventions could be faster, more engaging, and more appealing than face-to-face therapy for those who are more unwilling or resistant to social contact. Location-Based Games, on the other hand, can potentially bring hikikomori players out of their homes and lead to real-world social interaction [30,31]. Kato [31] even cites the case of a hikikomori patient who suddenly started going out to public places such as parks to play Pokémon GO, going so far as to interact with other players in the vicinity. This may be because of the nature of LBGs, coupled with an interest for the Pokémon franchise. Despite this suggestion, and the use of LBGs as a media for interventions in other populations such as the elderly [41], research that actively utilized LBGs to investigate the above claims at a broader level has yet to be conducted.

Alternatively, narrative-driven games like Visual Novels have also been suggested by Lu as a way to connect to hikikomori players via engaging stories and parasocial relationships [49]. This may be because of hikikomori people’s need to belong and desire to form some sort of connection, and those originating from fictional stories may be easier to maintain for this population. In a similar line of research, Panto and colleagues suggested that, by using fictional narratives like Visual Novels, a sense of empathy can be evoked from hikikomori players [50]. Their results showed a correlation between empathy and emotional transportation towards fictional narratives. They also found correlations between relaxation while consuming this media and fictional narrative consumption frequency, which suggests a possible association between fiction consumption and an empathy/relaxation response. However, further research on the topic, and on the reactions towards other game genres and elements, is yet to be conducted. Finally, past research has also proposed similar game-based intervention approaches that combine both visual novel and location-based elements.

## Methods

### Ethics statement

Participants were given detailed written instructions about the questionnaire presented and provided informed written consent by choosing to participate in the survey. This study was conducted following the Declaration of Helsinki and received approval from the Institutional Review Board of the Nara Institute of Science and Technology, with review number 2022-I-25.

### Survey

The survey consisted of a questionnaire with three sections: general sociodemographic questions, the NEET-Hikikomori Risk (NHR) Scale, and an open-ended questionnaire about gaming preferences. It was implemented using the SurveyMonkey platform, while the Crowdworks crowdsourcing website was used as the recruitment platform. We chose this approach because it is easier to reach hikikomori than through interviews. Hikikomori are traditionally avoidant of permanent employment or jobs involving social interaction, we reasoned that they would look for freelance or short-term, remote jobs, through crowdsourcing portals like Crowdworks. Moreover, the reclusive nature of the condition also suggests that, rather than conducting in-person or online interviews, open-ended surveys should be more effective in giving hikikomori an anonymous space to elaborate on their thoughts in a safe, controlled environment. To ensure the quality and facilitate the understanding of our results, the online survey was designed and reported in accordance with the CHERRIES checklist for web-based surveys [51]. An open, voluntary survey was conducted via the Crowdworks platform using a convenience sampling approach, targeting individuals aged 18 years or older residing in Japan with self-reported gaming experience. The questionnaire, implemented in SurveyMonkey, included the validated NEET-Hikikomori Risk (NHR) scale and open-ended questions on gaming preferences. Ethical approval was obtained prior to the study, and informed consent was obtained from all participants prior to participation. Data were collected anonymously, duplicate entries were screened via IP address checks, and incomplete responses were excluded from the final analysis. A detailed CHERRIES checklist [51] is provided in S1 Text.

### Participants

In total, 676 answers were collected from anonymous participants through the Crowdworks online platform in two rounds during February 2023. From these, 62 participants reported not having an interest in video games, four reported living outside of Japan, four declared not having been born and raised in Japan, and 72 did not complete the entire questionnaire. Furthermore, three gave answers that were considered “invalid” (e.g., writing random letters to fill up the answers), and two failed to correctly answer the attention-check question included in the middle of the questionnaire. Because the study pertained to gaming preferences, we specifically targeted people with gaming experience who self-reported interest in video games, who were above 18 years of age, born, raised, and currently residing in Japan. Therefore, the participants described above were excluded from the analysis, leaving a total of 529 participants after exclusion criteria ($M_{age}$ = 38.8, SD = 8.96; 50.47% men, 48.96% women, 0.57% chose not to answer).

To address the potential bias of convenience sampling and ensure the quality of our data, we implemented the validated NEET-Hikikomori Risk (NHR) Scale to precisely identify and isolate individuals at high risk of social isolation. By applying a predetermined 104-point cut-off score, we were able to distinguish between High-Risk (H-NHR) and Low-Risk (L-NHR) participants, effectively creating a “case-control” structure within our sample. This selection process resulted in a High-Risk group comprising 52.36% of our participants, confirming that we reached our target demographic. Regarding comparability, our sample (Mean age = 38.8; 50% male) aligns with contemporary Japanese research showing that the hikikomori phenomenon is no longer restricted to male youth but increasingly affects middle-aged individuals across genders [52].

### Measurements

The survey included general sociodemographic questions, attention-check questions embedded midway through to ensure participant attentiveness, the NEET-Hikikomori Risk (NHR) Scale [15], and questions about participants’ gaming preferences, dubbed **Game Preferences Survey.**

Based on a previous study, we used a 104 points cut-off for the NHR scores to distinguish between high and low risk NHR [53]. With this threshold, we found 52.36% of participants to be in the High NHR group, while 47.64% fall under the Low NHR group. The relatively high percentage of people in the high NHR group suggests that our assumption that hikikomori people would engage in crowdsourcing jobs is somewhat accurate.

#### NHR scale.

We decided to use the NHR Scale [15] to measure participants’ hikikomori risk tendencies as a psychological and motivational state, instead of choosing a more rigid form of classification that would require a formal psychiatric diagnosis based on behavioural profiles. This scale quantifies one’s risk of social isolation by measuring three main constructs: their uncertainty towards the future (e.g., “I don’t quite know what I want to do in the future”), their preference towards the NEET lifestyle (e.g., “I cannot find meaning in work”), and their feelings towards meeting societal expectations (e.g., “There are times when I think that I am not needed by society”). It measures participants’ agreement towards these statements using 7-point Likert scales.

According to Norasakkunkit & Uchida [15], a reliability analysis was done and showed that the scale’s combined items could represent an overall NEET-Hikikomori Risk, indicated by a Cronbach’s alpha of 0.82. Study 2, present in the same paper, also investigated and confirmed the validity of the scale, finding that NHR scores correspond with varying degrees of marginalized strata in a nationwide Japanese sample.

We categorized participants in high and low hikikomori risk, using a predetermined cutoff score of 104 points, previously established for the NHR Scale [53]. We looked for possible relationships between high and low NHR levels and game genres collected for the quantitative section of this study, as well as between NHR levels and game-related themes found in the qualitative section.

#### Game preferences survey.

Participants were asked to answer five questions with open-ended answers. In these questions, dubbed “Survey Questions” (SQ), participants were asked about the names of their favourite games, and what they liked about them. They were also asked their opinions about single-player, multiplayer, and online games. The translated Survey Questions can be seen in Table 1, while the original Japanese versions can be found in Table A of S2 Text.

**Table 1:** Game Preferences Survey - Survey Questions. Questions asked for both likes and dislikes on certain game types to get more opinions from participants.

Description
Please write the names of your favourite games.
Please write what features you like about each of your favourite games.
What do you like/dislike about single-player games?
What do you like/dislike about multi-player games?
What do you like/dislike about online games?

### Survey analysis

This study employed a mixed methods strategy to interpret participant answers. We separated the survey into two parts. The first part, dubbed **“Genre Analysis,”**, was subjected to quantitative bivariate analyses. In this section, we only asked participants for names of their favourite games. The goal was to extract game genres and keywords from game names, looking for possible discrepancies in terms of number of mentions of genres between high-risk and low-risk participants. Furthermore, we wanted to investigate the most mentioned genres from the high-risk group, as this could give us insight into the most popular game genres for this audience. This is also relevant when proposing features for game-related interventions, as it helps developers have a sense of the type of game elements they could focus on.

To extract game genres from game names, we used an online game database called RAWG API, which can be found at https://rawg.io. This API contains game-related information, collected via user contributions. It is currently one of the most maintained and extensive APIs on this topic. Because most of the game information is available in English, all participant answers were first translated into English. After this, we looked up the information on each of the participants’ chosen games in the API.

We chose the game from the RAWG API for each participant’s answer, aiming to choose the game that best fits participants’ reported games. If the participant reported a franchise instead of a single game (e.g., “Pokémon”, “Mario”), we chose the latest release of the main franchise that was present in the API (e.g., “Pokémon Scarlet & Violet”, “Super Mario Odyssey”). We avoided games outside of the main franchise unless explicitly named, since these games usually have a gameplay different from the main franchise (e.g., the main Pokémon franchise and Pokémon GO). There were occasions when we needed to adjust our data collection process. For instance, some participants wrote only genres instead of game names, such as “Puzzle”. In such cases, these were included in that participant’s genre list. Furthermore, we standardized some genre and keyword codes representing the same game feature (for example, “Side-Scroller” and “Side Scrolling”, or “Explore” and “exploration”).

Once the initial extraction was complete, the lead author thoroughly reviewed the genres and keywords collected to familiarise themselves with the data. The genres and keywords were then analysed to identify potential themes for grouping. After several iterations of the grouping process, a descriptive list of genres and keywords was created that preserved all relevant information. Furthermore, genres and keywords with less than ten instances were removed from the dataset, as their small frequencies led to incorrect approximations, and thus would not yield reliable conclusions. After this step, quantitative analyses were conducted, looking for relationships between the participants’ NHR Scores and the final list of genres and keywords.

With this data collected, we then conducted Pearson’s Chi-Square Test of Independence [54] to look for relationships between participants’ hikikomori risk levels and these genres and keywords. In cases where the frequency of the categories was too low for Pearson’s test to be reliably accurate, we used Fisher’s Exact Test [55] instead to confirm the significance of our findings. The continuous NHR Scores were categorised between “High NEET-Hikikomori Risk” (H-NHR) and “Low NEET-Hikikomori Risk” (L-NHR), according to Norasakkunkit’s cut-off calculation of 104 points [53]. All of the quantitative analyses performed in this study assumed an α value of 0.05 when determining statistical significance.

The second part of the analysis, dubbed **“Game Preferences Analysis,”** contained the four remaining questions (SQ2 through SQ5). These questions asked participants about what they liked in their favourite games. Furthermore, it asked about what elements of single-player, multiplayer, and online games were interesting and not interesting to these players. To examine these questions, inductive thematic analysis was employed to look for broader themes within participant answers, following Braun and Clarke’s approach [56,57].

In particular, we followed the phases of thematic analysis as described by Braun and Clarke [56], as well as a practical example of their methodology done by Byrne [58], adapting where necessary:

1. Data familiarisation: Three authors read the participants’ questions and answers, noting down initial ideas. Two authors had personal experience with playing video games in the Japanese context, and one had prior experience with thematic analysis. The coders had different backgrounds, including hardware engineering, software engineering and psychology research.
2. Generating initial codes: Based on their initial reading, authors created their individual themes. Themes connected with game elements and features were the primary goal of this analysis. Since the coders were not experts in game design, we used Sillaots, Jesmin, and Rinde’s list of game elements as a starting point [59]. This was used mainly to familiarise the coders with game-related terminology, such as PvP (“Player vs Player”). Nevertheless, coders were expressly told to use this list only as a guideline, focusing on extracting codes from the data instead.
3. Code standardisation: After coders had reviewed all the participants’ answers and created their own initial codes, they discussed these codes among themselves and sorted out any discrepancies or differences. For instance, while one coder might have used “RPG” to describe RPG-related game elements, another coder used “Role-playing elements,” so it was decided together to call such a code “Role-playing.” This process continued until a consensus was reached and the coders had arrived at a final agreed-upon, standardised code list.
4. Final coder agreement: from this list, we filtered the codes given to our participant answers. For each of the four questions in the Game Preferences Analysis, if the participant’s answers were coded with a specific code by at least two of the three coders the final version of the coded data would reflect that particular code for that answer.

Similar works in the literature approached the investigation of player gameplay preferences in a qualitative way, such as Rahman [60], which led us to decide on this approach. Braun and Clarke’s approach mainly describes the steps that a single coder can take to complete their thematic analysis. Given that we used three different coders, we adapted the original approach by including the “code standardisation” and “final coder agreement” steps. The use of multiple coders was done to minimise individual biases.

We also conducted a mixed-methods analysis using the results of this section, comparing individuals of high and low hikikomori risk on their responses towards certain codes.

## Results

### Genre analysis – Quantitative results

In total, 432 distinct game titles were collected from RAWG API, in a total of 1029 mentions. These yielded 1377 genre entries, composed of a pair of “Participant ID” and “genre mentioned,” and 5943 keyword entries. Participants also mentioned 69 distinct games, across 113 total mentions (10.98% of our total number of mentions pre-exclusions) that could not be found in the RAWG API. These were marked as “Not Available” and removed from the dataset. Many of these entries were either too niche to the Japanese market to be found in our chosen database, or too new to have detailed entries yet. Nevertheless, the remaining mentions still provide a adequate sample for this study.

When analyzing the data, 82 codes were also removed from the analysis. These included codes that were not in the English language, and codes that were considered not to have any impact on the gameplay itself or the perception of the game by players (for instance, “crowdfunded”).

From the 1377 genre mentions extracted from the players’ favorite games, we extracted 22 different genre categories. We also collected 616 original keywords from the 5943 keyword mentions. We converted all genres and keywords to the English language, grouped similar themes, and removed duplicated themes. Given that the Chi-Square Test is sensitive to sample size, a small sample size on some keywords could not yield reliable results for a robust analysis. Thus, we also removed keywords with frequencies lower than ten instances. The full list of rules used for keyword removal and merge can be found in S3 Text, along with the merged keyword list found in Table A of the same file. The final data frame contained 18 unique genres and 130 unique keywords.

**Fig 1:**
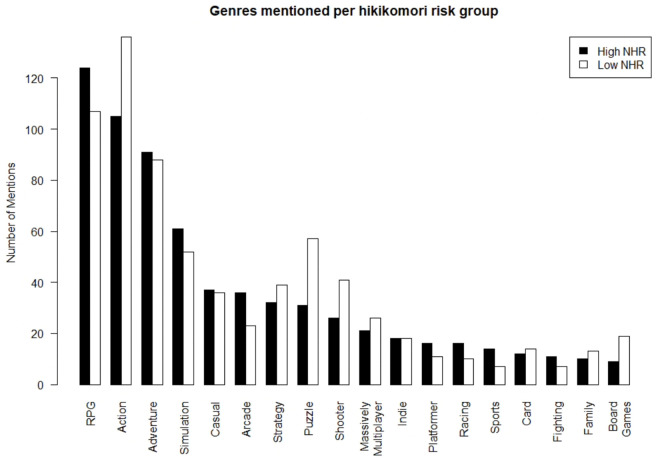
Mentions of game genres per hikikomori risk group. The image shows a bar plot of the number of mentions for each genre found in the quantitative analysis, separated between H-NHR and L-NHR groups, and ordered from most to least prevalent in mentions from the H-NHR group. RPGs, Action, and Adventure were the most frequently mentioned across both groups, followed by Simulation and Casual games. Action, Puzzle and Shooting genres were notably more mentioned by Low-Hikikomori Risk participants.

We executed the Chi-Squared Test on each individual genre and keyword to look for possible relationships between players’ hikikomori risk level and specific genres and keywords. The expected and observed values for all of the analysed Genres can be seen in Table A of S4 Text. The number of genre mentions for each group can be seen in Fig 1. To build the matrices necessary to calculate the Chi Square test for each genre and keyword, we separated the columns into High/Low Hikikomori Risk, and the rows into “mention and “no mention” for each genre. In other words, if a participant mentioned a favourite game that contained a certain genre (e.g., Action), then for the Action matrix, that participant would be included in the “mention” row, in the respective column depending on the participant’s NHR level. After applying the Benjamin-Hochberg correction procedure [61] to account for multiple comparisons, the bivariate analysis showed no significant relationship between NHR score and genres. There was also no significant relationship between NHR score and game keywords.

### Game preferences analysis - Qualitative results

After individually conducting the thematic analysis and discussing their results, the three coders and the lead author discussed the findings and agreed on a standardised code list of 92 codes.

Codes with high affinity were combined into 18 themes, which were further grouped into 4 super themes. The final groupings can be seen in S1 Fig. From these themes, we suggest possible game design elements and mechanics based on the qualitative analysis (Table 2). We also tied each theme and design suggestion to the SDT needs that it may evoke (Table 3). Below we go through each super theme and discuss each theme within.

**Table 2:** Game design requirements for high-risk hikikomori players. Requirements marked with * are non-functional requirements.

Design Suggestion	Source themes from Qualitative Analysis	Description
Short play sessions	Avoid physical Exertion, Freedom to explore and modify the game	Games that can be played for shorter periods of time, with small play sessions, to avoid physical strain and fatigue.
Short, diverse tasks	Freedom to explore and modify the game, Simple game mechanics, Relaxing repetitive tasks, Balancing good and bad replayability	Create small tasks of various kinds, which can be quickly accomplished over a small period of time. Make them diverse and non-repetitive to avoid player boredom.
Simple mechanics	Ease of play	Simple mechanics that can be easily picked up by many players.
RPG-like progression and mechanics	RPG-like Player Progression, Role-playing and Strategic Elements	Design elements that can show player growth and progression over time, such as level systems, skill trees, stronger character abilities, increase in earned in-game currency and items, access to new skills/weapons/areas, among others.
Mentally challenging puzzles	Role-playing and Strategic Elements	Design puzzle elements that make your players think and strategise in order to complete objectives (while taking into consideration the difficulty of the puzzles).
Involving, nostalgic world-building and narrative	World Building and Narrative, Immersion and presence are desired, Escapism from reality, Emotional well-being	When creating games with worlds full of experiences, craft well developed locations, characters and narratives that help transport the player to the game world, where they can temporarily be free from the stress of daily life. Touching on topics that evoke nostalgia, such as school life, could also help players connect with the story.
Deep and well-developed characters	World Building and Narrative, Immersion and presence are desired, Socialising is important	Create characters in the game that the players can get to know, socialise and possibly building bonds with.
Location-Based gameplay	Foster connections with faraway people/places	Creating gameplay that leads players outside, like Location-Based gameplay, could lead to a player connection to their environment. It could be used by players as they go about their daily commutes, and inversely, could help create habits of going outside to play.
High-paced action gameplay	Action gameplay	Design high-paced game modes that keeps players on their toes and focused on the action on the screen.
Customizable mechanics	Freedom to explore and modify the game (little time and design constraints)	Customizable mechanics to give the player the freedom to choose their play style, such as different abilities, appearances (skins) and playable characters (similar to Pokémon and Shin Megami Tensei series, where players choose monsters to fight for them).
Cooperative gameplay	Foster connections with faraway people/places, Socialising is important	Focus on cooperative gameplay over competitive, such as players working together to achieve some common goal where all can benefit from it. Cooperative gameplay may also lead to feelings of connectedness in players, which could be beneficial for their mental health.
Toxic behaviour management	Vocal about toxic interactions, Socialising is important	Control the way players can interact with each other, like limiting what they can say to each other, or having moderators paying attention to reports of negative behaviour and quickly taking action. Alternatively, design game mechanics that encourage good playing behaviour with positive feedback, such as asking players to rank the respectfulness of the other players after every match, and rewarding those who are ranked well with experience multipliers or other items.
Deliver complete experiences *	Vocal about game limitations	Create complete experiences from the beginning that can be played with little to no updates, or single-player experiences which do not require online servers to be maintained.
Data usage transparency *	Privacy issues	Manage player data properly and be clear about the usage of any data that is stored.
Avoid micro-transactions and pay-to-win mechanics *	Pay to win	Avoid including micro-transactions in the game, to avoid breaking game flow; avoid creating pay-to-win mechanics, which may generate feelings that the game is unfair towards players who don’t (or can’t) pay to become stronger.

**Table 3:** Mapping of game design requirements to the intrinsic needs of the SDT.

Design Suggestion	Autonomy	Competence	Relatedness
Short play sessions	X	X	
Short, diverse tasks	X	X	
Simple mechanics		X	
RPG-like progression and mechanics		X	
Mentally challenging puzzles		X	
Involving, nostalgic world-building and narrative			X
Deep and well-developed characters			X
Location-based gameplay			X
High-paced action gameplay		X	
Customizable mechanics	X	X	
Cooperative gameplay			X
Toxic behaviour management	X		X
Deliver complete experiences *			
Data usage transparency *	X		
Avoid microtransactions and pay-to-win mechanics *		X	

#### Physical and mental engagement.

This super theme is related to ways the game engages with the user and grabs their attention physically or mentally, be it in making the player physically move to play the game, or drawing the player’s attention by providing tasks and experiences that keep the player engaged.

**Avoid physical Exertion -** Respondents with high NHR scores disliked games that cause mental or physical fatigue, such as having to play for extended periods of time, or those which cause visual fatigue. This was particularly common among online game comments. One possible reason is that many online games, like MMO RPGs, require players to stay playing for long periods of time doing battles, quests, or other lengthy activities, often with other players involved synchronously.

*“Ensemble Stars! [I] have been playing it since before it was split into Music and Basic; I think the quality of Music is high, but I have been playing it somewhat less frequently because it requires me to keep my eyes on the screen.” (ID168, female, 35 years)*

**Relaxing repetitive tasks -** Players report enjoying tasks that can be done slowly, over time, such as being able to tend daily to small tasks. These tasks can distract them from daily life while not being too mentally taxing. The completion of simple tasks could foster the Competence intrinsic need of players. Being able to play whenever they feel like may also foster their Autonomy needs, as they are not bound to play at specific times.

*“It’s soothing to be able to escape the real world and relax in a fictional world. You can immerse yourself completely without thinking about anything else, and it helps you turn your eyes away from unpleasant things. ...” (ID306, female, 37 years)*

These tasks could also allow players to create healthy habits that foster positive feelings towards played games, improving the player’s mood.

*“...Harvest Moon: I like the fact that I can progress little by little every day and enjoy a relaxing farm life because it soothes me.” (ID300, female, 37 years)*

**Ease of play -** Some participants report that simple mechanics are fun or addictive to them.

*“Pacman is simple but addictive.[...]” (ID107, male, 52 years)*

They are also happy when they feel the game is balanced, as unfair advantages and disadvantages may throw off players (See “Pay to win” for examples of artificial imbalance). This ease of play and balanced mechanics could foster players’ Competence needs, as the balanced gameplay may lead them to feel competent when winning in their games.

*“I like that the story is good and also that the game is well-balanced and can be played for a long time.” (ID215, male, 48 years)*

**RPG-like Player Progression -** Players also reported enjoyment when feeling like they are progressing in developing their characters, items, or abilities. This feeling of progression could help fulfill their Competence needs, as numerical values in the stats of the players could serve as examples of progression in the game.

*“I like [games] that allow me to play thousands or tens of thousands of times. I like the fact that I can advance little by little every day and enjoy a relaxing farm life. Harvest Moon: I like that I can advance little by little every day and enjoy a relaxing ranch life.” (ID300, female, 37 years)*

*“...THE iDOLM@STER series: You are the producer and produce the idols, so you can enjoy the growth of the characters together.” (ID26, female, 30 years)*

**Immersion and presence are desired -** Some high-risk hikikomori players report liking the immersive experience that well-developed worlds and narratives can provide. As they interact with characters in the game world, this sense of immersion and connection with a new world full of unique characters could foster feelings of Relatedness.

*“Twisted Wonderland: The story is very interesting, and the unique characters and worldview are wonderful. There are elements of battles and rhythm games, but I also enjoy getting myself into the world and progressing through the story.” (ID255, female, 54 years)*

**Balancing good and bad replayability -** Replayability enhances a game’s shelf life, making players spend much time playing them. Some features that developers can implement to increase a game‘s replayability include collectibles, achievements, and in extreme cases, making the game not have a concrete ending, instead allowing the player to endlessly play it. Games that never end create mixed feelings in players. Some may feel frustrated because there seems to be no end goal on sight.

*“I hate it because it never ends” (ID73, female, 41 years)*

On the other hand, some players like the idea of a game never-ending, as they can always have something to do in that game.

*“The endless development of the game allows you to pass the time as much as you want, which I think is one of its strengths.” (ID148, male, 30 years)*

When a game inevitably reaches the end, this can leave some players frustrated. In these cases, using procedurally generated quests could increase the game’s lifespan.

*“(I like to do things leisurely, such as making things and watering plants, without being restricted by time. I dislike it when I have nothing to do after a certain routine is finished.” (ID202, female, 38 years)*

Even so, games may inevitably end or become boring to players, even if they have many quests and collectibles to go through.

*“[In] Final Fantasy 14 [...] there was a lot of work to do the same things over and over again, and I got bored after about two years. Skyrim, Animal Crossing, Stardew Valley, and Cookie Clicker could be played at my own pace and as I wanted, so I could play them and play them for a long time. These were also my favourite games, although I did get bored with them.” (ID266, female, 41 years)*

Despite all these measures to increase replayability, it is important to remember that a player’s interest in any game will most likely end at some point. Therefore, it is important to understand this finitude when creating game-based interventions, and accepting that a single game will most likely not serve as a never-ending intervention approach.

**Freedom to explore and modify the game (little time and design constraints) -** Many participants cherish the ability to play the game at their own pace and in short amounts at a time. This could help foster players’ Autonomy needs, as they get to decide when and how much they want to play at any given time.

With the advent of technology, studies have found that higher social media use was consistently related to poor attentional control and higher distractibility [62]. This may lead some gamers who extensively use social media to not be able to focus for long periods of time in video-games. Because of this and the fact that social media usage is prevalent with hikikomori individuals [47], some hikikomori players may enjoy games that can be picked up for short amounts of time when they have free time.

*“Skyrim, Animal Crossing, Stardew Valley, and Cookie Clicker could be played at my own pace and as I wanted, so I could play them for a long time. …” (ID266, female, 41 years)*

*“You can proceed slowly at your own pace.” (ID217, male, 41 years)*

Other players report liking the freedom to explore the game world they are in or to customise their characters and abilities to their liking.

*“Animal Crossing: New Horizons: You can design your island and house as you like, and visit your friends’ islands via communication. The animals in the game are also cute.…” (ID26, female, 30 years)*

*“Stray: A very innovative exploration game with a realistic cat as the main character. The story of a lost cat exploring a mysterious world in order to return home to his family and discovering what happened in the past in the world with cats was fascinating. ” (ID25, female, 43 years)*

#### Positive and negative consequences of game usage for player.

This super theme relates to problems and positive sensations that arise from playing the game, such as being frustrated by game limitations or grateful for the out-of-reality experience and escapism that some games might provide.

**Escapism from reality -** Some players show positive feelings about games that allow them to experience a different, perhaps kinder, reality than the one they are used to. Some games achieve this through world-building, which helps temporarily transport the player to an alternate reality where they can leave their real-world worries behind while playing.

*“Animal Crossing: I enjoy being able to live in a different world. I wanted to live in such a world.” (ID40, female, 25 years)*

In contrast, some players report that emulating a different reality can help them relieve stress by getting them into situations otherwise impossible in the real world. This escapism mechanism could offer some catharsis to players and be a healthy way of helping participants deal with the stress of daily life.

*“I like GTA series because I can relieve stress by doing things I can never do in real life. ” (ID69, male, 40 years)*

**Vocal about game limitations -** Some players report frustration over game downtime, be it because of updates, or occasional server maintenance that stops them from playing their games.

*“It is bad that there are periods [in online games] when it is unavailable due to maintenance.” (ID45, male, 35 years)*

**Privacy issues -** Some online game players report feeling like they’re being watched while they are playing, which could be a consequence of the current society’s increasingly blurry line between private and public information, especially in social media. Players may also feel as if companies are holding onto their data for uses that were not agreed upon.

*“I sometimes feel uncomfortable because I feel as if someone is always watching my movements.” (ID463, male, 45 years)*

*“I don’t like the part where I feel like I am constantly being watched.” (ID488, female, 37 years)*

**Pay to win -** Players voice disliking having to pay to have a fighting chance in games, because the levelling curve is too slow/steep or because other players have paid to become stronger. Games that have these features can hinder the fulfilment of players’ Competence needs, as an unfair or pay-walled progression curve may make players feel like they are not good at the game.

*“I think the biggest advantage is that you can enjoy the game without being alone and lonely. What I don’t like is that I can’t keep up with the level of users around me and I have to pay for the game. Also, it seems to be difficult to find members who fit in with you.” (ID150, male, 52 years)*

#### Gameplay elements and features.

This super theme focused on specific gameplay elements mentioned by participants.

**World Building and Narrative -** Not all games need narrative elements, but if they are included, focusing on interesting narratives and captivating characters could create positive experiences for high-risk hikikomori players. Players frequently make positive mentions of well-developed narratives and fleshed-out characters that they have experienced in their favourite games.

*“Final Fantasy 14 had an interesting storyline and was the most enjoyable online game I have played in a long time.” (ID266, female, 41 years)*

*“Fire Emblem series: All of the games are high quality SRPGs, and it’s a lot of fun to twist your brain frantically and create your own strategies and battles. The characters are attractive, and the support conversations are a great way to learn about unexpected aspects of the characters.” (ID49, female, 30 years)*

These parasocial relationships could help foster their Relatedness needs, as Lu [49] points out, which could help as part of the resocialization process by introducing a benign relationship to socially isolated players.

*“Kingdom Hearts: I’ve played all of the series, and it’s a lot of fun because it’s linked to the Disney story, has original elements as well, and uses Disney characters in a nice way. ” (ID134, female, 34 years)*

**Role-playing and Strategic Elements -** Role-playing games are one of the oldest video game genres, originating in classic tabletop RPGs. Many high-risk hikikomori players stated their interest in RPG mechanics and gameplay, and how being able to strategise within such games made them feel. These mechanics often include improving your characters through battles and raising their levels and abilities, while progressing through the game narrative.

*“I enjoy steadily catching Pokémon every day and raising their level.” (ID85, female, 37 years)*

*“Dragon Quest, Final Fantasy, and Xenogears are famous RPGs that you can enjoy levelling up.” (ID175, female, 39)*

Similarly, other games that require strategic thinking, such as puzzle games, were also well mentioned by this audience, who seemed to be happy with having mental challenges in their games.

*“Poco Poco is a puzzle game, but I like it because it gives me a sense of satisfaction when I clear the game and when I get the anticipation right.” (ID114, female, 46 years)*

*“The puzzle game is fun and the events are challenging to collect special characters and furniture.” (ID28, female, 40 years)*

These games can provide challenges that, when conquered, could foster players’ Competence needs.

**Action gameplay -** High-paced action elements such as thrilling battles and exciting competitions were described as interesting by some players.

*“I like Nier because of its action and thought-provoking story.” (ID69, male, 40 years)*

*“God of War: With its heavy story and high action, I remember playing it in one sitting, especially attracted by the story.” (ID25, female, 43 years)*

Being able to clear difficult action-packed sequences in games could help with players’ Competence intrinsic needs.

*“The Legend of Zelda - It’s full of puzzle-solving and action elements, and the music and story are also highly polished, so you can get immersed in the world of the game.” (ID10, male, 32 years)*

#### Social relationships and their management.

The positive and negative aspects of maintaining social relationships within gaming, and how this vision may change within different gaming modes, such as cooperative vs. competitive games were also mentioned.

**Emotional well-being -** Players report liking situations where they feel satisfied when playing a game that reminds them of older games of the same series, or reminds them of situations from their past, like their schooldays. While nostalgia for older entries in a franchise is difficult to create when creating a brand new game, appealing to elements from the common past of players, such as relatable feelings related to common childhood life, such as school life and being surrounded by family members, could help in creating a feeling of nostalgia. This nostalgia could help players feel like they belong in that moment they are experiencing, and could help foster their Relatedness needs.

*“Dragon Quest of the Stars: At first, I felt nostalgia for the story and music. Now I want to strengthen characters and equipment, become stronger, and enjoy battles. ” (ID255, female, 54 years)*

*“Tokyo Majin Gakuen series: The scenario is well written and you are drawn into the story more and more. The dialogue between friends and characters is fun to play and makes you feel like a student again.” (ID49, female, 30 years)*

**Foster connections with faraway people/places -** Despite the stereotype that hikikomori people are anti-social, reports from high-risk hikikomori players show a different side to the story; in online games, players seem to cherish the connection with others, feeling as if they were forming relationships with other people. These relationships, even with those who are physically distant, could help players with their Relatedness needs.

*“I like the fact that I am connected to many different people online, which gives me the feeling of having many friends. ...” (ID437, male, 45 years)*

Going out and exploring one’s environment could also be a good way for players to foster connections to their immediate locations. Some high-risk hikikomori players even reported positive experiences when going out to play Location-Based games such as Pokémon GO and Dragon Quest Walk.

*“[Dragon Quest Walk] makes me want to go out of my way and enjoy walking, which is very enjoyable.” (ID216, female, 42 years)*

*“Pokémon Go is great because you can play it when you are out and about.” (ID483, male, 42 years)*

**Fostering socialization, favouring cooperation** - Many players report positive feelings of socialising in games in many forms, be it casually, cooperating towards goals, or competing with one another.

*“It’s like a multiplayer game, you can play and compete with many different people, so it’s hard to get bored and it’s fun and exciting. I like the fact that there are more and more contents to be added through updates and other means.” (ID389, male, 35 years)*

Even socialising with non-playable game characters can be seen as interesting for some participants, who reported enjoying the socialising elements in some games combined with other game features.

*“Harvest Moon… Interesting multitasking where you not only manage the farm but also communicate with the residents.” (ID40, female, 25 years)*

While cooperation and competition are both valid forms of social interaction, the latter seems less interesting to high-risk hikikomori players.

*“I don’t like to be in competition with others.” (ID50, female, 60 years)*

*“I don’t like [multiplayer games]...Cooperative play is okay, but competing with someone in a game is hard.” (ID40, female, 26 years)*

*“I like being able to play in a guild or party. I also like the fact that there are many games where you can freely change your avatar. I don’t like it when people [kill my character] while I’m not playing.” (ID228, male, 56 years)*

Cooperating with others, be them real players or parasocial relationships with NPCs, could help players feel like they belong to a group by joining together towards a common goal, which could fulfill some of their Relatedness needs.

**Vocal about toxic interactions between people -** Unfortunately, social gameplay could also have negative consequences, as intense and difficult gaming can frustrate players. Playing with others, especially in a competitive environment, can lead to players taking out their frustrations on each other.

High-hikikomori players were also vocal about their fear of being a nuisance when playing with others. One participant mentioned how they were worried about making mistakes during interactions with teammates.

*“Final Fantasy 14 had an interesting storyline and was the most enjoyable online game I have played in a long time... however, I did not enjoy the high difficulty content because it required interaction with others and I was worried about my own mistakes.” (ID266, female, 41 years)*

Other high-risk hikikomori players report being frustrated with the toxic interactions with players, and how they were unable to do anything to prevent or stop such behaviour and had to resort to waiting for moderators to deal with the issue.

*“[In online games,] It’s easy to meet people you don’t like and it’s hard to avoid them. The downside is that it is up to the management to deal with them.” (ID389, male, 35 years)*

## Discussion

### On the relationship between hikikomori risk and gaming preferences (RQ1)

When analysing the most mentioned genres, our quantitative results showed that Action, RPG, Adventure, and Simulation were the four genres most present in the games reported by participants in both groups. This is in line with research done by Kubo et al., which showed that Role-playing, Puzzle, Action, Simulation, and Adventure games were the most popular genres played by hikikomori players [17]. The fact that most of these genres appear in both studies points towards the interest in these game genres by hikikomori audiences as well. This is in and of itself a relevant point to warrant further research and take into account when developing games for these audiences.

In Fig 1, it can be seen that Action, Puzzle and Shooter genres were more commented by participants in the Low risk group. This could be related to the inherent difficulty present in games of these genres, which may cause some disinterest from high-risk hikikomori players. In past research, Norasakkunkit & Uchida [53] compared how Japanese students with different levels of hikikomori risk responded when presented with negative feedback after challenging tasks. They found that people with higher hikikomori risk were less likely to persist with the challenging task when confronted with negative feedback compared to low-risk students. A similar outcome could happen when high-risk hikikomori players are faced with difficult games that are hard to beat. This could explain why challenging games that require problem-solving and fast reflexes could lead to less engagement by those in the H-NHR group, as repetitive failure could lead to player disengagement.

To statistically test these descriptive tendencies, we executed bivariate analyses, but despite the observable trends, our analysis did not reveal a statistically significant relationship between hikikomori risk and game preferences, described by genres and keywords. Nevertheless, the descriptive results point toward gameplay preference patterns that are consistent with prior literature [17], indicating that preferences for genres like Action and Puzzle are relevant for both low- and high-risk players. Considering this and the qualitative results, where some high-risk participants also reported liking action and puzzle game mechanics, we can see that such game genres may still be appreciated by high-risk hikikomori players, although to a somewhat lesser extent because of possible issues with handling constant negative feedback [53].

While our results, particularly in the quantitative front, point towards descriptive tendencies that are consistent with prior literature [17], it is important to acknowledge that our interpretation of the qualitative data is unavoidably shaped by the background of our research team. The coding of qualitative answers and overall interpretation of the results was conducted by researchers with backgrounds in hardware engineering, software engineering, and psychology research. This multidisciplinary perspective could have influenced the themes emphasized during analysis, particularly when interpreting differences between quantitative trends and qualitative reports. We therefore caution that the comparisons done between qualitative and quantitative findings should be seen as interpretative rather than contradictory.

### On the relationship between hikikomori risk level and playing with other people (RQ2)

Unsurprisingly, we noticed differences in the way players in L-NHR and H-NHR reacted to playing with others. For instance, high-risk players pointed out how they enjoyed playing with others, but, at the same time, reported problems with toxic players and their inability to deal with it in many cases.

Similarly, High NHR level players preferred collaboration over competition. One possible reason for this is a general aversion towards player interactions. Yong & Nomura [63] found that interpersonal difficulties were the most significant and strongest indicator for hikikomori. Hikikomori participants seem to feel anxiety about interpersonal relationships, and Yong & Nomura state that “these anxieties may be related to a sense of humiliation, which suggests that they are afraid of being seen in their current situation.” In competitive Player-vs-Player games, where emotions are high and there is a lot of tension and sometimes toxicity between players during matches, it could be the case that hikikomori players may feel afraid of the social consequences of losing matches, such as how their opponents or even their own teammates might treat them in the face of a defeat. Because of this, high-risk hikikomori players could be more averse to challenging situations when facing other skilled humans in a competitive manner, similar to the results of Norasakkunkit & Uchida [53], which found that high-risk hikikomori students were less likely to persist in challenging tasks. This could be further exacerbated when considering reports of problems with “pay-to-win” mechanics, where other players pay money to become artificially stronger, which can further cause imbalances between players in matches.

In summary, while some high-risk players seem to enjoy player interactions, they are more biased towards cooperative aspects, as competition can bring about toxic behaviour and feelings of unfairness caused by differences in player skills and pay-to-win mechanics. This result is similar to findings from Mihardi et al. [19], where players claimed to enjoy social interactions caused by multiplayer gaming experience, but also reported in-game chats as a frequent source of toxic interactions.

### On game genres and features that may affect hikikomori player engagement (RQ3)

According to our results, the H-NHR group seemed less interested in the social aspects of video games, particularly when such aspects involve connections with other players. They were also more vocal when it came to negative player interactions. These players still display interest in having people with whom they can share fun moments and accomplishments with. However, the introduction of complicated human relationships seems to be a problem for some.

To account for negative interactions between players, such as harassment, which is particularly mentioned by the H-NHR group, games could have a stronger oversight on player interaction, utilising techniques such as sentiment analysis to filter out rude communication between players. Another way to tackle this issue is by limiting the types of messages players can send to each other, so that negative messages can be reduced or even removed entirely.

Another approach that does not rely on communication filtering could instead take the form of positive reinforcement. Games like League of Legends and Dawngate have implemented honour systems towards this goal. In these systems, players are asked to rate their teammates and opponents at the end of matches, and those who are rated well by all players get more experience bonuses and bonus items. With this system, players will be more inclined to be nice to each other to get more bonuses, which could potentially solve issues with player toxicity.

In terms of which player interaction could be the most engaging for hikikomori players, player competition seems to be less attractive to players with higher hikikomori levels. One possible reason is that competition instils the idea that there will always be a winner and a loser, which could, in turn, lead to the aforementioned problems of harassment and rude player interactions. This, coupled with previously related problems of player toxicity and “pay-to-win” mechanics, could be the cause of less interest in competition from the H-NHR group. Therefore, game developers who wish to include social interactions in hikikomori games might wish to focus on more cooperative gameplay.

Despite the apparently less frequent mentions of action and puzzle elements by H-NHR players (see Fig 1), the qualitative analysis did show that such elements can and are still enjoyed by some in this group. One possible reason for this apparent contradiction between the quantitative and qualitative results may lie in the difficulty setting of these games. While action and puzzle elements, in general, may still be enjoyable for high-risk hikikomori players, the high difficulty present in some games may cause high-risk players to give up more easily than low-risk players. This can cause high-risk players to still like these game genres, but only actually play games that are less difficult, or that provide ways to alter their difficulty setting.

Players in both groups mention well-developed and engaging world-building, narratives, and characters. Past research has studied the relationship between hikikomori people and story-driven Visual Novels. Lu has previously mentioned that hikikomori people may enjoy Visual Novels due to their intrinsic need for connection with others [49]. Similarly, Panto and colleagues studied the relationship between hikikomori and empathy through the use of Visual Novels [50]. It is possible that hikikomori players enjoy the use of interactive stories and interesting characters because of a desire to have some sort of relationship with others, while at the same time retaining control over how much they are willing to emotionally invest in such a relationship. Although the use of narratives and fictional characters does not replace real human connections, nor does it solve the problem of loneliness that some players may face, it could be used as a first step for players more averse to real human connections. By incorporating newer technologies such as large language models and chatbots such as ChatGPT (https://chat.openai.com), game developers could create more complex characters and engaging narratives. These interactions may improve a player’s self-perception, or their perception of relationships with others.

When discussing game genres that may appeal to hikikomori audiences, the results point to games with high-paced action or strategic elements, such as action, role-playing, strategy, puzzle, and adventure games.

This is also backed up by previous research [17], which found that Role-playing, Puzzle, Action, and Adventure were among the genres most played by hikikomori players. Taking this into account, game developers could implement fast-paced action gameplay elements in games catered to hikikomori players, while properly balancing the difficulty setting of these elements, so that hikikomori players who are averse to hard challenges don’t become demotivated to continue playing. Alternatively, such games could also benefit from strategic, logical puzzle-like elements, such as enigmas for the player to solve, or classic turn-based RPG battle systems, for example.

Finally, some in the H-NHR group still report enjoying going out and playing location-based games. In such cases, going out to play is the main goal for some, while for others, it is a consequence of having to go out and wanting something to do during their commute. This could be an interesting angle for game developers to explore when looking for ways to lead hikikomori people out of their homes more frequently. By creating games that incorporate the real world in their gameplay, players must go out to accomplish tasks, and in the process might reconnect to their surroundings, or even have some interaction with people outside or other players that they might encounter.

While the proposed design implications (e.g., short play sessions or RPG-like progression) may be relevant for engaging individuals with higher NEET-Hikikomori Risk, it is important to note that the present study does not evaluate whether such engagement is beneficial or detrimental. In the broader literature, distinctions between healthy and problematic gaming are typically based on behavioural outcomes such as loss of control and functional impairment, rather than on specific game mechanics alone. Moreover, operationalizing “healthy” engagement would require assessing clinical and behavioural indicators beyond the scope of the present study. For example, prior research on randomized reward systems (“loot boxes”) has shown associations with problem gambling severity and gambling-related cognitions [64,65], with evidence suggesting that these relationships may be stronger in contexts involving real-world monetary expenditure [67,66]. Therefore, the implications proposed here should be interpreted as exploratory and hypothesis-generating.

Importantly, this study focuses on identifying engagement patterns within a population at higher risk of social withdrawal, representing a necessary first step in the design of game-based interventions. Without initial engagement, such interventions are unlikely to be adopted or sustained. Therefore, our findings highlight high-isolation risk player preferences and sensitivities, which can inform the development of systems that are both engaging and accessible to this population. Future research should examine how these design elements can be implemented within frameworks that promote healthy and adaptive gaming behaviours.

### Recommendations for design

We suggested game mechanics suitable for hikikomori players based on our results (see Table 2). Game elements that seem less attractive to high hikikomori players mainly include social aspects and features. These features could still be implemented in games for hikikomori audiences, but developers should be careful when designing player interaction mechanics (see “Filter and manage toxic social behaviour” design suggestion for further explanation).

While the identified genres (Action, RPG, etc.) provide a broad overview of the gaming landscape favoured by hikikomori players and corroborated by past research [17], the practical utility of these findings lies in the identification of categories that can be broken down further into specific characteristics of each genre or broader interchangeable features useable in any of them. This can then be translated to needs-based design requirements grounded on the SDT. We propose a layered design approach, where these broad game genres serve both as the foundational “vessels” for interventions, and as a basis for future studies that can investigate particular characteristics of each genre. This foundation must then be refined by mapping each genre’s inherent strengths and characteristics to the intrinsic needs of the SDT (Autonomy, Competence, Relatedness), while applying the specific functional and non-functional requirements identified in our qualitative analysis (see Table 2). For example, a broad “Action” or “RPG” vessel is practically translated into an effective intervention for the H-NHR group by implementing “RPG-like progression” and “simple mechanics”, to fulfill Competence needs. This specific implementation ensures that players experience a sense of growth without the high-difficulty hurdles or negative feedback that typically lead to disengagement in high-risk individuals. Similarly, “short play sessions” can be implemented across all preferred genres to fulfill Autonomy by allowing players to manage physical and visual fatigue at their own pace. Furthermore, social mechanics within these genres should be steered toward “cooperative gameplay” to meet Relatedness needs, while bypassing the social anxiety and toxic interactions reported by this demographic. By layering these specific, qualitative requirements onto familiar genres, developers can create game-based interventions that are both engaging and accessible to a population sensitive to social rejection and high difficulty.

## Conclusion

This study used mixed methods analysis to investigate whether the risk of becoming hikikomori correlates with an individual’s gaming preferences. With these results, we sought to understand the game design implications from such findings, in order to propose game elements and features for game-based interventions towards high-risk hikikomori players. Using the Self-Determination Theory, we categorized each design requirement based on its contribution to fulfilling each of the three intrinsic needs described by this framework (Autonomy, Competence, and Relatedness). We found requirements that could influence a player’s Autonomy needs, such as short play sessions with customizable, short, and diverse tasks. Players’ Competence needs could be fulfilled by design elements like simple mechanics, RPG-like progression, and mentally challenging puzzles. Finally, players’ Relatedness needs could benefit from mechanics such as deep and immersive world-building and narrative, location-based gameplay, and toxic player behaviour management. By using these design requirements, game developers can design better game experiences for these players, both for commercial purposes and also for game-based interventions that can be a first step towards behavioural change, aiming to bring hikikomori people back to a more social lifestyle. While video games have shown promising results in the past in helping players with their mental health [18], and past research has shown the potential of using this media to reach socially isolated audiences [27,28], such as using location-based features to lead people outside and foster socialization [32,33], it is important to reiterate that game-based interventional approaches are not meant to substitute traditional intervention approaches such as therapy, but rather to complement them and potentially be used as an initial intervention towards resocialization.

Quantitative analyses did not find significant differences in gaming genre preferences of high- and low-risk hikikomori participants. Despite this, our findings show that the general preferences for high-risk hikikomori individuals align with previous research that investigated hikikomori players’ gaming preferences [17], with their favourite games falling mostly within the Action, RPG, Adventure, Simulation, and Puzzle genres. While this suggests that, genre-wise, developing games for players with high risk of hikikomori may not be much different from developing for the general public, the similar findings among research solidify these highly mentioned genres as popular ones that may lead to higher player engagement.

Qualitatively speaking, we found that those with a higher risk of becoming hikikomori enjoy games where they can play at their own pace (which can help fulfill the player’s intrinsic motivation of autonomy), games with fast-paced action, engaging narratives, and strategic elements.

When it comes to social game features, H-NHR players seem to have strong negative experiences related to player harassment in-game, and seem to prefer cooperative features over competitive ones. In contrast to L-NHR players, those in the H-NHR group showed less interest in the social aspects of games. This aligns with the antisocial aspects often present within the hikikomori condition. Nevertheless, we also observed an interest in interaction with other players. This could suggest that, despite some aversion to player interaction, hikikomori players are still interested in it. It is possible that their aversion stems mostly from the fear of bad interactions or being judged by others, rather than a simple lack of interest in communicating with other players.

Finally, location-based elements, while having few mentions, were often depicted in a positive light by H-NHR players. Given that one of the defining features of these games is making players roam around the real world as a core game mechanic, they could also be used in game development for high-risk hikikomori, particularly for game-based interventions that wish to bring players out into the real world.

One of the limitations of the current study was the lack of information sources related to more niche Japanese games played by some participants. Despite this being a relatively small percentage of mentioned games, future studies on the topic could benefit from using other sources of information to account for games specific to Japanese audiences, in order to better understand the games consumed mainly by this group of players. This can lead to a more precise understanding of the game preferences of Japanese hikikomori audiences, and subsequently new game design suggestions.

Furthermore, while the quantitative analysis found no significant results in the current study, there seems to be some discrepancy in players’ opinions related to some game genres, such as Action and Puzzle, in terms of number of mentions and qualitative opinions. Further research with multivariate analysis is warranted to properly investigate the relationship between the hikikomori condition and specific game genres, as well as the possible rationale behind such relationships.

Given that our primary focus was the possible relationship between NEET-Hikikomori Risk and gaming preferences, this study did not assess Internet Gaming Disorder, gameplay duration, or other indicators of problematic gaming behaviors. Therefore, while the game design suggestions offered for high-risk players may be relevant in building player engagement for this group, no conclusions can be drawn regarding whether the identified game features promote healthy or unhealthy engagement. Future studies should investigate how specific game design features interact with gameplay duration and behavioural outcomes, to better distinguish between adaptive and maladaptive engagement patterns.

Another potential improvement for this study is the expansion of the scope of participants to include other countries and cultures. Despite the hikikomori phenomenon no longer being considered a culture-bound syndrome to Japan, individual game preferences may vary across different cultures. Similarly, much like the alignment between this study and the findings from Kubo et al. [17], game genre preferences among hikikomori individuals may align with those from the general public, but further research is needed to validate this hypothesis.

Finally, while we did acknowledge the relationship between the hikikomori condition and ASD [12,14], we could not assess autistic traits among participants of this study. Considering this, future work should examine whether game preferences could also be affected by players’ autistic traits, helping to clarify whether certain game genres and features are more or less suitable based on different neurodiversity profiles.

## Supporting information

S1 Fig Qualitative Analysis - Separation of codes and themes. (TIF)

S1 Text CHERRIES Checklist (Checklist for Reporting Results of Internet E-Surveys). (PDF)

S2 Text Research Questions - Original Japanese version. (PDF)

S3 Text Game Genre Analysis - Rules for removing and merging keywords. (PDF)

S4 Text Game Genre Analysis - Number of mentions found of all genres. (PDF)

S5 Text Thematic Analysis Codes - Number of mentions and mean emotions for L-NHR and H-NHR groups. (PDF)
