# Supplementary figures and images for: Extreme social isolation risk is associated with story-driven, strategic and cooperative-first gameplay preferences
Source: PLOS Ment Health. 2026 Jul 29;3(7):e0000517. doi: 10.1371/journal.pmen.0000517 (PMC13419178; doi:10.1371/journal.pmen.0000517)

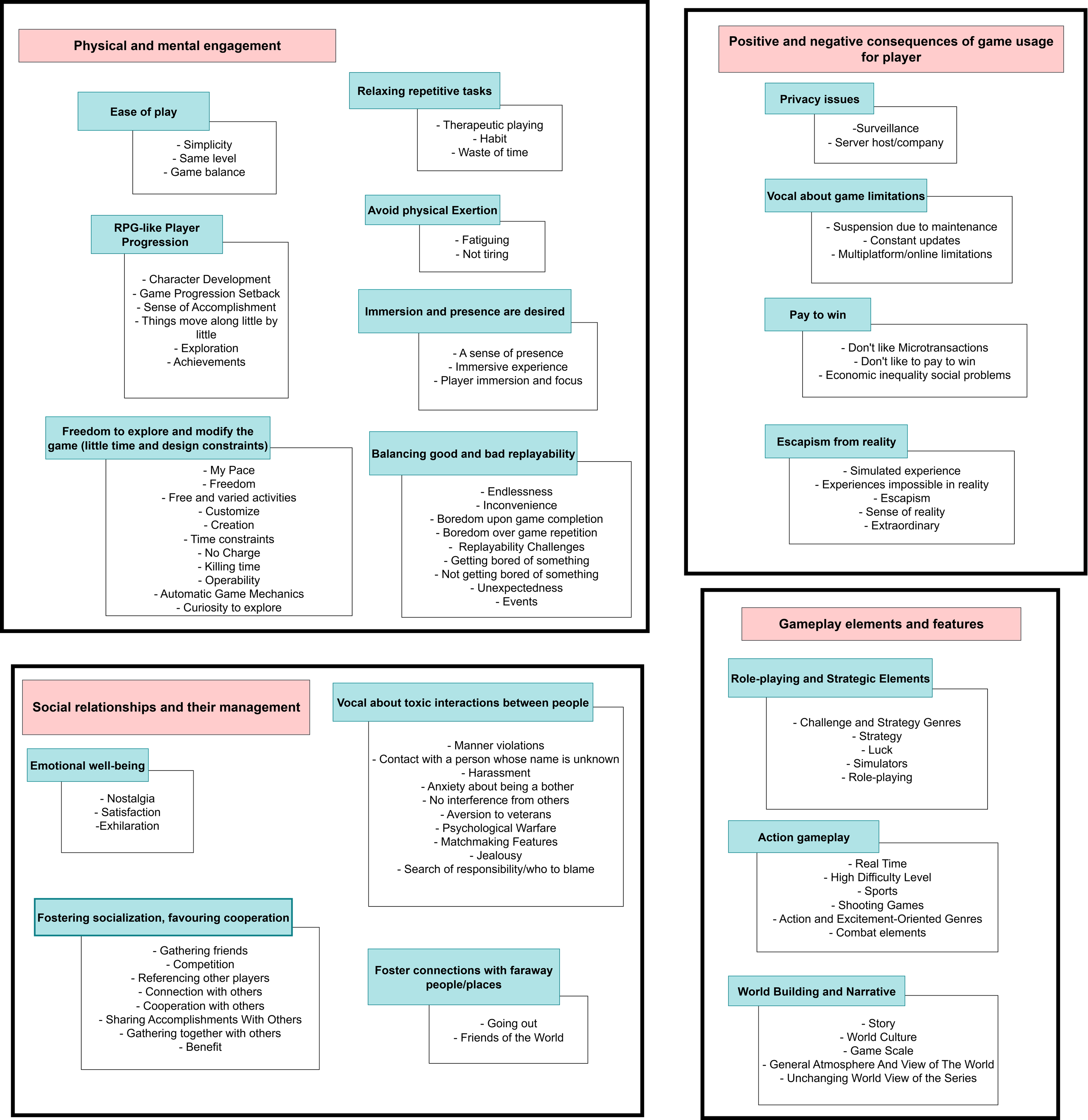

Supplement: S1 Fig — (TIF) [file pmen.0000517.s001.tif]
