# Supplementary material for: Extreme social isolation risk is associated with story-driven, strategic and cooperative-first gameplay preferences
Source: PLOS Ment Health. 2026 Jul 29;3(7):e0000517. doi: 10.1371/journal.pmen.0000517 (PMC13419178; doi:10.1371/journal.pmen.0000517)
Supplement: S1 Text — (PDF) [file pmen.0000517.s002.pdf]

S1 Text. CHERRIES Checklist (Checklist for Reporting Results of Internet E-Surveys)

1) Design

Target Population: People with gaming experience who self-report interest in video games, aged 18+, born, raised, and currently residing in Japan.

Survey Type: An open survey (accessible via the Crowdworks platform to any user meeting the criteria) with a convenience sample.

Technical Platform: Implemented using the SurveyMonkey platform.

2) IRB Approval

IRB Approval: Approved by the Institutional Review Board of the Nara Institute of Science and Technology (Review Number: 2022-I-25).

Informed Consent: Detailed written instructions were provided prior to the start of the survey; participants gave informed written consent by choosing to participate.

Data Protection: The survey was anonymous. IP addresses were collected automatically by the SurveyMonkey platform but were not used or disclosed during any part of the study.

3) Development and Pre-testing

Development: The survey used the validated NEET-Hikikomori Risk (NHR) Scale and customized open questions about participants' interests in certain types of games.

Pre-testing/Pilot: The survey was manually pilot-tested by the lead author before full deployment.

4) Recruitment Process and Sample Description

Open vs. Closed Survey: Open survey through Crowdworks (a Japanese crowdsourcing/freelance website).

Contact Mode: Internet, conducted in two rounds during February 2023.

Advertising the Survey: Advertised through the Crowdworks platform.

5) Survey Administration

Mode: Web-based survey.

Context: Crowdworks is Japan's largest online crowdsourcing platform, connecting companies with freelancers and individuals for various work tasks. It was used to recruit participants for this survey.

Participation: Voluntary.

Incentives: Monetary reward of 500 JPY upon completion of the questionnaire.

Time/Date: Conducted in two rounds during February 2023.

Randomization: The order of questionnaire items was not randomized.

Adaptive Questioning: Descriptive questions about favorite games and opinions on single/multiplayer games were only presented if participants answered "Yes" to the initial question ("Do you play games?").

Number of Items: The questionnaire contained between 40 and 50 items, depending on participant responses.

Number of Screens: Not recorded.

Completeness Check: Conducted using R; incomplete responses were excluded from the final analysis.

Review Step: Participants were able to review and modify their answers before final submission.

6) Response Rates

Unique Site Visitor: Crowdworks collected IP addresses as metadata. These were used to check for duplicate entries; none were found.

View Rate: Not available.

Participation Rate: Not available.

Completion Rate: A total of 676 participants agreed to participate, of which 72 did not complete the survey. The completion rate was 89.3%.

7) Preventing Multiple Entries

Cookies: Not used.

IP Check: IP addresses were collected as metadata and used to detect duplicate entries; none were found.

Log File Analysis: Not used.

Registration: Not used.

8) Analysis

Handling of Incomplete Questionnaires: Incomplete questionnaires were excluded from the analysis.

Atypical Timestamps: Not assessed.

Statistical Correction: Not applied.
