# Supplementary material for: Extreme social isolation risk is associated with story-driven, strategic and cooperative-first gameplay preferences
Source: PLOS Ment Health. 2026 Jul 29;3(7):e0000517. doi: 10.1371/journal.pmen.0000517 (PMC13419178; doi:10.1371/journal.pmen.0000517)
Supplement: S2 Text — (PDF) [file pmen.0000517.s003.pdf]

S2 Text. Research Questions -Original Japanese version

Table A: Game Preferences Survey - Japanese Questions

Question ID	Description
**Q1**	好きなゲームの名前を書いてください（たくさん書いても構いません）。
**Q2**	好きなゲームの感想をそれぞれお聞かせてください。
**Q3**	シングルプレイヤーゲームについて、どんなところが好き/嫌いですか?
**Q4**	マルチプレイヤーゲームについて、どんなところが好き/嫌いですか?
**Q5**	オンラインゲームについて、どんなところが好き/嫌いですか?
