# Supplementary material for: Extreme social isolation risk is associated with story-driven, strategic and cooperative-first gameplay preferences
Source: PLOS Ment Health. 2026 Jul 29;3(7):e0000517. doi: 10.1371/journal.pmen.0000517 (PMC13419178; doi:10.1371/journal.pmen.0000517)
Supplement: S3 Text — (PDF) [file pmen.0000517.s004.pdf]

S3 Text. Game Genre Analysis - Rules for removing and merging keywords

This section explains how we decreased the size of the keyword list used in the Game Genres Analysis, by removing codes that do not pertain to gameplay elements for some reason, and merging others who fit a bigger, common theme.

Rules for code removal:

1. **Languages other than English:** To avoid redundant keywords that are already represented in our final list, we removed any non-English keywords.

2. **Random Objects with low frequency:** random keywords like 'flowers', 'cubes', etc, that may be part of the theme of a game, but not necessarily translate to gameplay preferences, and that had frequencies smaller than ten mentions, were removed, as they would not yield reliable results with the quantitative analysis used. Similarly, tags such as 4X or 'c', which did not have a clear meaning, have been removed.

3. **"Non-functional requirements":** Features that are related to what could be considered a non-functional requirement in software development, for instance, "cloud saves". Also includes "game states" that do not translate to the actual game experience, like "alpha", "beta", "crowdfunded". EXCEPTION: Codes about developed platform (iOS, Android, etc) were kept, as they could yield relevant information.

4. **Vague adjectives:** Adjectives that do cannot be easily translated to something related to the gameplay or visuals and are too vague to represent any particular group of gameplay elements. For instance, "Beautiful" and "Difficult" can be related to gameplay mechanics (graphics and challenging gameplay), but 'amazing' may not be, as games can be amazing in many different ways.

5. **Specific franchises or companies:** Codes like "Nintendo", "Sega", "Zelda" etc., while being able to give an general idea of gameplay elements from brand identity, are too broad to offer useful information to our analysis.

Rules for code merging:

1. **Codes that represent elements but also mention genres:** Examples like "2D" and "2D Fighter" should just become "2D". The "Fighter" part is a genre, and is already represented in the "Genres" part of the analysis, thus becoming redundant here.

2. **Plural and singular words:** For instance, merging "plant" and "plants" into "plants".

3. **Keywords with similar meanings, or that evoke one bigger theme:** Examples include merging "spells", "wizards", "alchemy" and "Magic" into "Magic-related Elements", or "ship", "naval", "sea", "Pirates", "water", "wave" and "waves" into "Sea-related Elements".

List of non-English removed keywords:

Acquisti dall'applicazione, Achievement di Steam, Cartes à échanger Steam, Compat. total con mando, Compatibilidade parcial com controle, Comquistas Steam, Compras em aplicativo, cooperativo, cooperativo online, Giocatore singolo, JxJ (Jogador x Jogador), Logros de Steam, multijogador, Succès Steam, Supporto Completo per i controller, Um jogador, Un joueur, Un jugador

List of removed keywords for other reasons:

1980s, 1990', 4X, addictive, alpha, awesome, beta, amazing, America, baby, bundl, c, camera, captions available, cloud saves, controller, controller support, crowdfunded, community, disney, doom, Early Access, european, exclusive, GitHub, in development, in-app purchases, Kickstarter, Ludum Dare, mario, Mary Jane, masterpiece, meta, nintendo, other, overlay, pack, Pokémon, pong, Remote Play on Tablet, Remote Play on TV, Remote Play Together, Sega, shining-series, Sonic, Steam Achievement, Steam Cloud, Steam Leaderboard, Steam Trading Card, Steam Workshop, steam-trading-card, tobey-maguire, true exclusive, tv, Valve Anti-Cheat Enabled, wip, youtube, Zelda.

Table A: Game Genre Analysis - Merged Keywords

**Original Keywords**	**Final Keywords**
2D, 2D Fighter	2D
3D, 3D Platformer	3D
android-game, android-games, android-mobile	Android
animal, animals	Animal
ar	Augmented Reality
app, mobile, mobile-game, phone	Made for Mobiles
arcade-classic, arcade classic	Arcade
arena, arena Shooter	Arena
ancient, ancient-greece	Ancient Setting
atmosphere, Atmospheric	Atmospheric
ball, balls	Balls
tower, Tower Defense, defense	Tower Defense
alternating turns, Turn-Based, Turn-Based Combat, Turn-Based Strategy, Turn-Based Tactics, turn-based-battle	Turn Based Combat
Beat 'em up, beat-up	Beat 'em Up
block, blocks	Blocks
board-game-simulation, board-games, Board Game	Board Game
bug, bugs	Bugs
Building, build, construct	Building
car, cars	Cars
Cartoon, Cartoony	Cartoon
cat, cats	cats
Character Customization, character-creation, character-generator	Character Customization
character, characters	characters
choice, Choices Matter	Choices Matter
city, City Builder	City Builder
Class-Based, Classes	Class System
click, Clicker	Clicker
Co-op, Co-op Campaign, co-op multiplayer, coop, co-operative, Local Co-Op, Online Co-Op, Shared/Split Screen Co-op	Cooperative Gameplay
Online PvP, Shared/Split Screen PvP, competition, Competitive	Competitive Gameplay

coin, coins	coins
collect, collection, collectathon	Collectibles
color, colors, Colorful	Colorful
Controller, controller support	Controller Support
Dark, darkness, Dark Comedy, Dark Fantasy	Dark
destroy, Destruction	Destruction
dungeon, Dungeon Crawler	Dungeon Crawler
e-sports, esports, sport, Football, Soccer	Sports
Exploration, explore	Exploration
Agriculture, farm, Farming, Farming Sim	Farming Simulator
fast, Fast-Paced	Fast-Paced
fight, fighter	Fighting
first person mod, first person shooter, First-Person, first-person shooter, FPS	First Person
3rd-person, 3rd-Person perspective, Third Person, Third-Person Shooter	Third Person
flower, flowers	flowers
fly, flying, Flight	Flying
fun, Funny, humor, Memes	Funny
future, futurism, Futuristic	Futuristic Environment
girl, girls	Girls
Hack and Slash, hack-n-slash	Hack 'n Slash
adult, Mature	Mature Audiences
eroge, hentai, Sexual Content, NSFW, Nudity	Sexual Content
hero, Hero Shooter	Heroes
Historical, history	Historical Elements
hunt, hunting	Hunting Elements
immersive, Immersive Sim	Immersive Simulation
ios, ios-game, ios-games	Apple (iOS)
japan, japanese	Japanese
Local Multiplayer, Multiplayer, multiplayer-only, Online multiplayer, Split Screen, Cross-Platform Multiplayer	Multiplayer
JxJ, JxJ online	JxJ (Jogador x Jogador)
CRPG	RPG (CRPG)
JRPG	RPG (JRPG)
mmo, MMORPG	RPG (MMO)
Role Playing Game, role-playing, RPG	RPG
Tactical, tactics	RPG (Tactical RPG)
kid, kids, kid-safe, Kids/Family, Family Friendly, safe-for-kids	Family Friendly/For All Ages
life, Life Sim	Life Simulator
Loot, lootboxes	Lootboxes
Lore-Rich, Story, Story Rich	Game Story
Management, Resource Management	Resource Management
mary jane, mary-jane	Mary Jane

match, matching	Match-3 Mechanics
mine, Mining	Resource Collection
Mod, moddable, modding	Has Mods
Narration, Narrative, Dynamic Narration	Narrator
nintendo-switch, console	Made for Consoles
Open World, open-world	Open World
planet, planets	planets
plant, plants	plants
Platform	Platformer
procedural-generation	Procedurally Generated Content
puzzles	Puzzle Elements
race, racer	Racing
Mechs, Robots	Robots
run, Runnner	Endless Runner
ship, Naval, sea, Pirates, water, wave, waves	Sea-related Elements
shoot, shooter-game	Shooting Mechanics
Singleplayer, Single player only	Singleplayer
sound, soundtrack, Great Soundtrack, music	Soundtrack
student, school	School-Related Elements
spells, wizards, alchemy, Magic, witch	Magic-related Elements
Survival, Survival Horror, survival-horror-game	Survival Elements
tank, tank controls, Tanks	War Tanks
Team, Team-Based	Team-Based
text, Text Based	Text Based
VR, vr mod	VR Elements
War, Wargame, World War II ,ww2, army	War-themed
Drama, Emotional	Dramatic
friendly, friends	Friends
Investigation, Detective	Investigation Mechanics
Dating Sim, Romance	Romantic Elements
murder, crime, Violent	Violence Elements
peaceful, non-violence	Non-Violence Elements
steam-trading-cards, Steam Trading Cards	Steam Trading Cards
