# Supplementary material for: Extreme social isolation risk is associated with story-driven, strategic and cooperative-first gameplay preferences
Source: PLOS Ment Health. 2026 Jul 29;3(7):e0000517. doi: 10.1371/journal.pmen.0000517 (PMC13419178; doi:10.1371/journal.pmen.0000517)
Supplement: S4 Text — (PDF) [file pmen.0000517.s005.pdf]

S4 Text. Game Genre Analysis - Number of mentions found of all genres

Below, we can see all the genres found in the quantitative analysis. They were found by extracting the genres of the favourite games of each participant, using the RAWG API.

Table A: Genre Analysis - Mentions per NHR group

Genre	Mentions	H-NHR (O)	H-NHR (E)	H-NHR (O)/(E)	L-NHR (O)	L-NHR (E)	L-NHR (O)/(E)
Action	241	105	117.52	89.35	136	123.48	110.14
RPG	231	124	112.64	110.09	107	118.36	90.40
Adventure	179	91	87.29	104.25	88	91.71	95.95
Simulation	113	61	55.1	110.71	52	57.9	89.81
Puzzle	88	31	42.91	72.24	57	45.09	126.41
Casual	73	37	35.6	103.93	36	37.4	96.26
Strategy	71	32	34.62	92.43	39	36.38	107.20
Shooter	67	26	32.67	79.58	41	34.33	119.43
Arcade	59	36	28.77	125.13	23	30.23	76.08
Massively Multiplayer	47	21	22.92	91.62	26	24.08	107.97
Indie	36	18	17.55	102.56	18	18.45	97.56
Board Games	28	9	13.65	65.93	19	14.35	132.40
Platformer	27	16	13.17	121.49	11	13.83	79.54
Card	26	12	12.68	94.64	14	13.32	105.11
Racing	26	16	12.68	126.18	10	13.32	75.08
Family	23	10	11.22	89.13	13	11.78	110.36
Sports	21	14	10.24	136.72	7	10.76	65.06
Fighting	18	11	8.78	125.28	7	9.22	75.92

L-NHR = Low NHR, H-NHR = High NHR, (O) = Observed, (E) = Expected , (O)/(E) = Percentage of Observed/Expected
