# Supplementary material for: Extreme social isolation risk is associated with story-driven, strategic and cooperative-first gameplay preferences
Source: PLOS Ment Health. 2026 Jul 29;3(7):e0000517. doi: 10.1371/journal.pmen.0000517 (PMC13419178; doi:10.1371/journal.pmen.0000517)
Supplement: S5 Text — (PDF) [file pmen.0000517.s006.pdf]

S5 Text. Thematic Analysis Codes - Number of mentions and mean emotions for L-NHR and H-NHR groups

Below are the results for all mentions of each code present in the final version of the thematic analysis data. It is separated by group (H-NHR and L-NHR) and shows percentage of mentions per group, along with each groups emotion mean for that code (-1 being purely negative mentions, 1 being purely positive mentions).

Table A: Codes - Percentage of mentions and mean emotion per NHR group. EM - Emotion Mean; % L-NHR = percentage of mentions from the Low NHR group; % H-NHR = percentage of mentions from the High NHR group)

**Code**	**Mentions**	**%H-NHR**	**%L-NHR**	**EM H-NHR**	**EM L-NHR**
Self-sufficient	5	100	0	1	N/A
Fad/trend	4	100	0	-1	N/A
Sharing accomplishments with others	4	100	0	1	N/A
Benefit	3	100	0	1	N/A
No interference from others	3	100	0	1	N/A
Referencing other players	3	100	0	1	N/A
Game Scale	2	100	0	1	N/A
Gathering friends	2	100	0	0	N/A
Psychological Warfare	2	100	0	1	N/A
Suspension due to maintenance	2	100	0	-1	N/A
Voice actor	2	100	0	1	N/A
Advertisement	1	100	0	-1	N/A
Boredom upon game completion	1	100	0	-1	N/A
Change to third person perspective	1	100	0	1	N/A
Extraordinary	1	100	0	1	N/A
Feeling of emptiness	1	100	0	-1	N/A
Habit	1	100	0	1	N/A
Inferiority complex	1	100	0	-1	N/A
Not tiring	1	100	0	1	N/A
Self-responsibility	1	100	0	1	N/A
Vehicles	1	100	0	1	N/A
Inconvenience	14	85.71	14.29	-1	-1
Endlessness	6	83.33	16.67	-0.6	1
Sports	15	80	20	1	1
Simulated experience	5	80	20	1	1
Nostalgia	8	75	25	1	1
Surveillance	4	75	25	-1	-1
High difficulty level	10	70	30	0.14	0.33
Exploration	13	69.23	30.77	1	1
Getting bored of something	13	69.23	30.77	-1	-1
Harassment	42	66.67	33.33	-1.00	-1
Nervousness	15	66.67	33.33	1	1
Unexpectedness	6	66.67	33.33	1	1
Server host/company	3	66.67	33.33	-1	-1
Shooting Games	3	66.67	33.33	1	1
Vulnerability	3	66.67	33.33	-1	-1
Self-efficacy	19	63.16	36.84	-1	-0.71
Multiplatform/online limitations	34	61.76	38.24	-0.81	-0.85

Gathering together with others	102	60.78	39.22	1.00	1
Music	20	60	40	1	1
No charge	20	60	40	1	1
Luck	5	60	40	1	-1
Manner violations	32	59.38	40.63	-1	-1
Challenge and Strategy Genres	29	58.62	41.38	1	1
Freedom	53	58.49	41.51	1.00	1
Game Content	12	58.33	41.67	1	1
Strategy	44	56.82	43.18	1.00	1
Character Statues	101	56.44	43.56	1.00	1
Combat elements	16	56.25	43.75	1	1
Sense of reality	16	56.25	43.75	1	1
Microtransactions	18	55.56	44.44	-1	-1
Aversion to veterans	9	55.56	44.44	-0.6	-0.5
Curiosity	9	55.56	44.44	1	1
Matchmaking features	98	55.1	44.9	-0.96	-1
Player immersion and focus	82	54.88	45.12	0.96	0.78
Story	86	54.65	45.35	1.00	1
Events	22	54.55	45.45	1	1
Time constraints	24	54.17	45.83	-1	-1
Loneliness	13	53.85	46.15	-1	-1
Fatiguing	17	52.94	47.06	-1	-1
Connection with Others	126	52.38	47.62	0.52	0.63
Role-Playing	23	52.17	47.83	1	1
Update	25	52	48	1	1
Not getting bored of something	37	51.35	48.65	1.00	1
Cooperation with others	103	50.49	49.51	1.00	0.96
My Pace	391	50.38	49.62	1.00	1
Achievements	16	50	50	1	0.75
Waste of time	8	50	50	-1	-1
Automatic Game mechanics	4	50	50	1	1
Boredom over game repetition	4	50	50	-1	-1
Escapism	4	50	50	1	1
Monotony	4	50	50	-1	-1
Game balance	2	50	50	1	1
Same Level	2	50	50	1	1
Search for responsibility/who to blame	2	50	50	-1	-1
Game Elements	56	48.21	51.79	1.00	0.93
Sense of accomplishment	23	47.83	52.17	1	1
Operability	17	47.06	52.94	1	0.56
Creation	15	46.67	53.33	1	1
Satisfaction	11	45.45	54.55	1	1
Action and Excitment-Oriented Genres	20	45	55	1	1
Jealousy	20	45	55	-1	-1
Character Development	27	44.44	55.56	1	1
Therapeutic playing	18	44.44	55.56	1	1
Replayability challenges	25	44	56	1	1
Unchanging world view of the series	16	43.75	56.25	1	1
General atmosphere and view of the world	35	42.86	57.14	1	1
Education	7	42.86	57.14	1	1

Anxiety about being a bother	19	42.11	57.89	-1	-1
Going out	12	41.67	58.33	1	1
Exhilaration	67	40.3	59.7	1.00	1
Real Time	10	40	60	1	0.67
Game Progression setback	33	39.39	60.61	-1	-1
Graphics	39	38.46	61.54	1.00	1
Simplicity	39	38.46	61.54	1.00	1
Killing time	16	37.5	62.5	1	1
Things move along little by little	8	37.5	62.5	1	1
Friends of the World	39	35.9	64.1	1.00	1
Contact with a person whose name is unknown	29	34.48	65.52	-1	-1
Competition	42	33.33	66.67	1.00	1
Experiences impossible in reality	3	33.33	66.67	1	1
Simulators	3	33.33	66.67	1	1
A Sense of Presence	4	25	75	1	1
Customize	4	25	75	1	1
Free and varied activities	4	25	75	1	1
Immersive experience	6	16.67	83.33	1	1
Anytime	3	0	100	N/A	1
Main character	2	0	100	N/A	1
PvP	2	0	100	N/A	1
Bugs	1	0	100	N/A	-1
No replayability challenges	1	0	100	N/A	-1
Offline	1	0	100	N/A	1
