# Supplementary material for: Extreme social isolation risk is associated with story-driven, strategic and cooperative-first gameplay preferences
Source: PLOS Ment Health. 2026 Jul 29;3(7):e0000517. doi: 10.1371/journal.pmen.0000517 (PMC13419178; doi:10.1371/journal.pmen.0000517)
Supplement: Attachment — Submitted filename: Response to Reviewers.docx [file pmen.0000517.s007.docx]

**For editorial board and reviewers:**

We thank you for your careful consideration of our manuscript and your useful comments. In this letter, we provide point-by-point responses to those comments, addressing questions and problems pointed out by the reviewers. The original reviewer comments will be marked in **bold**, while the responses will be marked in blue. Additions included directly in the manuscript will be marked in red here.

--------------------------------------------------------------------------------------------------------------------

**Reviewer #1:**

**I appreciated the opportunity to review your article. I thought that your writing was well reasoned and easy to follow. I have some fundamental concerns about seeing a gaming intervention meeting the criteria laid out for SDT. I understand and don't necessarily disagree that there could be short-term and limited benefit in the SDT categories of autonomy, competence, and relatedness. However, my concern is that the article seems to suggest that this would be an overall viable intervention approach, but I don't see how this translates to something concrete in the real world. I don't see the leap from potentially improved online interaction and isolated improved mood to a functional social being. I think that using gaming as a potential therapeutic response drives us further into a dystopian realm where reality based human interaction becomes increasingly unlikely.**

Thank you for your comment. Our research team is involved in related research programmes where social skills interventions are conducted through video games (and gamified systems) on Minecraft for hikikomori youth. While research is still ongoing, preliminary results show some significant improvement in mental health and qualitative improvement in hikikomori symptoms. While we cannot discuss this in the manuscript as the research is ongoing, we think there is potential for games to be used as mediums for therapeutic interventions, by meeting the patients (in this case, hikikomori youth) at the platforms where they may be comfortable. While not on hikikomori directly, research has shown how video games can improve mental health (such as <https://dl.acm.org/doi/abs/10.1145/3411763.3451608>), and using location-based games can be helpful towards building relationships not only in a virtual world, but also in real life (see <https://link.springer.com/chapter/10.1007/978-3-030-15616-9_8>, <https://onlinelibrary.wiley.com/doi/abs/10.1111/ssqu.12880>). Finally, it is important to reiterate that the current research proposes the use of video games to help with socialization as a potential first step to aid hikikomori audiences, but it is not argued that is should nor is able to substitute traditional approaches such as therapy, but instead serves as a complement to them. This point has been further clarified in the Introduction and Conclusion sections.

**I do think your research should be published, however, in my opinion I think some commentary on whether this could ever empower a person to successfully improve their mental health outside of the virtual context would be appropriate.**

Thank you for your comment. Past research has shown that games have the potential to improve mental health of players (see <https://link.springer.com/article/10.1186/s12889-024-21011-9>). In the social aspect, games not only have the potential to create online relationships and help with loneliness and social anxiety (see [www.sciencedirect.com/science/article/pii/S0747563215302508](http://www.sciencedirect.com/science/article/pii/S0747563215302508)), but could also lead to offline social support (see [www.sciencedirect.com/science/article/pii/S0747563211002706](http://www.sciencedirect.com/science/article/pii/S0747563211002706)). In this sense, while the proposed game-based approach in this manuscript focuses on the possible improvement of socialization through game media (and not outside of it), there is potential for this media to bring about positive mental health and socialization consequences to the real world. These findings were added to our Introduction, and show the potential of using game-based interventions for socialization.

**Reviewer #2:**

**I enjoyed reading your work, though do consider some overarching features need revision / expansion (see uploaded response to your paper).**

Thank you for your careful comments in the uploaded response. We have analyzed the points made and adjusted the paper accordingly. Please refer to the highlighted portions of the updated manuscript.

**You might also consider whether there is a conflict of interest (being game designers yourselves). [...] and a statement on positionality is also required.**

Thank you for your comment. Our department’s main focus is on Information Science, broadly speaking, and in particular, on extended realities. We are not game designers, and we have corrected the sections of the paper that might give this impression. Furthermore, the research team is composed of psychologists and engineers. The coder’s background was mixed, including a hardware engineer, a software engineer, and a psychology researcher. This was included in the Data Treatment sub-section.

The coders had different backgrounds, including hardware engineering, software engineering and psychology research.

Therefore, we consider that there were no conflicts of interest.

**There is a need for some coverage of neurodiversity, that you hint at, and beyond this, you might consider that making gaming more suitable for those who exhibit H-NHR may be critiqued too. The real world might not be so accommodating, and so some consideration of how gaming might for example encourage some robustness or resilience as a narrative seems required, to demonstrate a purpose, otherwise the work might be critiqued for encouraging further entrenchment to isolation in H-NHR people.**

Thank you for your comment. We have added some coverage on neurodiversity in the Introduction, showing the current findings on the relationship between hikikomori and comorbidities such as Autism Spectrum Disorder:

Other comorbidities, such as Autism Spectrum Disorder (ASD), have also been perceived in hikikomori participants. In a pilot case–control study from Katsuki et al., hikikomori participants were more likely to have higher autistic tendencies, based on the Autism-Spectrum Quotient questionnaire. Similarly, Brosnan & Gavin have shown that higher levels of autistic traits could serve as a mediator to increased hikikomori risk in young adults. These findings suggest links between the hikikomori and ASD, making it more difficult to understand the limits and properly characterize the hikikomori phenomenon and its separation from other previously established conditions.

About making the game suitable for H-NHR players, as the reviewer points out, the real world is usually not so accommodating, and thus creating a game tailored to high-risk hikikomori individuals would not perfectly reflect the real world. However, our reasoning is precisely that such games can be more accommodating to players than the real world, and therefore offer a better first experience towards resocialization, by controlling the environment and interactions and allowing for possible filters of negative interactions.

The goal of using game-based interventions is not to perfectly mirror the real world through a gamification lens, but to be able to shape and control the way that players interact with and experience the world through that medium. To properly help those who are socially isolated, we believe that it is first necessary to provide a safe psychological environment to do so, which may be in the form of video games. One reason for this is the potential for using anonymized avatars, which can protect users’ self esteem and help them integrate into connected communities, improving players’ mental health (see <https://ojs.aaai.org/index.php/ICWSM/article/view/19351> for an example of this approach). Furthermore, virtual environments such as Massively Multiplayer Online (MMO) games have been shown to be useful for providing spaces for social interaction, which can help in the formation of bridging social capital (<https://academic.oup.com/jcmc/article/11/4/885/4617703>, <https://www.sciencedirect.com/science/article/pii/S0747563215302508>). Finally, the use of virtual reality as a therapeutic approach specifically for hikikomori individuals has also been suggested in the literature, showing the potential of this medium in helping those who are socially isolated (<https://journals.sagepub.com/doi/10.1177/00207640241270748>). The examples above showcase how, by shaping the way that users interact with reality and with others, media such as VR and games can be used as a first step towards resocialization. As mentioned previously, this approach is not meant to substitute traditional approaches such as therapy, but rather to complement them. Thus, by combining a “softer”, safer interaction with their environment and others, coupled with traditional approaches, hikikomori people could be slowly reintroduced into society in safer ways. This could also decrease the risk of blowback and bad social interactions early in the resocialization process, which could also lead to “further entrenchment to isolation” that the reviewer points out. Consistent with this view, past research (<https://doi.org/10.3389/fpsyt.2023.1133659>) has also showed efficacy of video-game based interventions for hikikomori when used in creating the initial rapport between the hikikomori and social workers/clinicians as a base for further therapies and treatments.

**There also seems to be an error in reporting the chi square in the general discussion,…**

Thank you for your comment. The chi square mentions for relationships between genres/keywords and hikikomori risk was from a previous version of the manuscript and is incorrect. We have removed this mention from the updated manuscript. Furthermore, the methodology for the Chi Square tests done on individual genres and keywords was further explained for clarity.

To build the matrices necessary to calculate the Chi Square test for each genre and keyword, we separated the columns into High/Low Hikikomori Risk, and the rows into ``mention and ``no mention'' for each genre. In other words, if a participant mentioned a favourite game that contained a certain genre (e.g. Action), then for the Action matrix, that participant would be included in the ``mention'' row, in the respective column depending on the participant’s NHR level.

After applying the Benjamin-Hochberg correction procedure to account for multiple comparisons, the bivariate analysis showed no significant relationship between NHR score and genres. There was also no significant relationship between NHR score and game keywords.

We also corrected our findings in the Discussion regarding the mistaken quantitative results from previous versions. Given the findings and their relationship with existing literature, we clarified these results in the updated “On the relationship between hikikomori risk and gaming preferences (RQ1)” subsection, and reiterated this as a limitation of the current study that warrants further research into the topic:

Furthermore, while the quantitative analysis found no significant results in the current study, there seems to be some discrepancy in players' opinions related to some game genres, such as Action and Puzzle, in terms of number of mentions and qualitative opinions. Further research is warranted to properly investigate the relationship between the hikikomori condition and specific game genres, as well as the possible rationale behind such relationships.

We apologize for this oversight, and have corrected it accordingly.

**… and an appreciation of the mental health implications seems warranted here too.**

Thank you for your comment. As explained above, while the quantitative results in the current manuscript did not find a significant relationship between hikikomori risk and game genres, from the number of mentions displayed in Figure 1 in the manuscript, we can notice a certain trend towards certain genres such as Action and Puzzle being more favoured by low-hikikomori risk players, which could be explained by the difficulty setting present in these genres (see <https://spssi.onlinelibrary.wiley.com/doi/abs/10.1111/j.1540-4560.2011.01727.x> for the rationale behind this). We have reflected this in the manuscript accordingly in the updated “On the relationship between hikikomori risk and gaming preferences (RQ1)” subsection.

**Some clarity on the use of self determination theory as you progress through your paper would be good also, as might expansions on behavioural and cognitive components you mention.**

Thank you for your comment. In the manuscript, there is a table where we summarize each relevant design suggestion, and how it possibly relates to each construct in the Self-Determination Theory. For clarity, we have also expanded the Analysis subsection of the Qualitative Analysis, including explanations on how each theme present in our thematic analysis could relate to each relevant construct presented in the Self-Determination Theory. For instance, the following was added to the “Relaxing repetitive tasks” theme:

The completion of simple tasks could foster the Competence intrinsic need of players. Being able to play whenever they feel like may also foster their Autonomy needs, as they are not bound to play at specific times.

For other examples of where the Self-Determination Theory may apply to each theme, please check the highlighted parts in the referenced subsection.

**Clearer link to mental health literature with regards to your introduction, thematic analysis and recommendations seems warranted too.**

Thank you for your comment. We have included additional literature on the suggested topics, particularly in the Introduction regarding the general mental and physical benefits of gaming (see [bmcpublichealth.biomedcentral.com/articles/10.1186/s12889-024-21011-9](http://bmcpublichealth.biomedcentral.com/articles/10.1186/s12889-024-21011-9)), as well as the social aspect of gaming (see [https://www.sciencedirect.com/science/article/pii/S0747563211002706](https://www.sciencedirect.com/science/article/pii/S0747563211002706?casa_token=llfWMxOcWvkAAAAA:KW0UTul1Kn59EWqx15NGtI5JlAzjoiR51LdGUka-xq_QAh7KgkNJTR1Q-pK9SyxaseQPjQC8rus) as an example). We have also included literature to support that games can be used as social spaces, which could support their use as a “first step” approach towards resocialization (see [https://www.sciencedirect.com/science/article/abs/pii/S0747563215302508](https://www.sciencedirect.com/science/article/abs/pii/S0747563215302508?utm_source=chatgpt.com))

On the neurodiversity aspect that the reviewer has pointed out, we have introduced literature pertaining to the relationship between hikikomori and Autistic Spectrum Disorders (see <https://onlinelibrary.wiley.com/doi/full/10.1111/pcn.13154>, <https://journals.plos.org/plosone/article?id=10.1371/journal.pone.0281833> for examples) and acknowledged that relationship in the Introduction. Since our focus during the investigation was on hikikomori risk levels, we did not assess autistic traits on the participants, and thus added this limitation at the end of our manuscript for future works.

Finally, while we did acknowledge the relationship between the hikikomori condition and ASD, we could not assess autistic traits among participants of this study. Considering this, future work should examine whether game preferences could also be affected by players' autistic traits, helping to clarify whether certain game genres and features are more or less suitable based on different neurodiversity profiles.

**A fuller consideration of H-NHR and gaming disorder seems apt, and a consideration that gaming might be a component to be managed, so that real social interaction might be encouraged also, for greater wellbeing. The omission of such arguments may prevent a balanced appraisal of the needs for those who exhibit Hikikomori. Your work is intriguing, though honing it for a clinical audience would benefit your wish to publish it here.**

Thank you for your comment. The relationship between the hikikomori condition and increased Internet usage/internet gaming disorder has been described in previous studies (see <https://journals.lww.com/co-psychiatry/abstract/2020/05000/internet_society,_internet_addiction,_and.14.aspx>, [https://www.sciencedirect.com/science/article/pii/S2352853218300877](https://www.sciencedirect.com/science/article/pii/S2352853218300877?utm_source=chatgpt.com), <https://pmc.ncbi.nlm.nih.gov/articles/PMC11488624>). This relationship has been explained in the Related Works section.

Regarding gaming management and social interaction encouragement, while real social interaction is the final goal for approaches that aim to help socially isolated individuals, the goal of the current paper is to understand gaming preferences of people at high risk of becoming socially isolated. With this understanding, game-based interventions, such as serious games and gamified applications could be developed in the future for this particular audience.

We believe that game-based interventions can be used as a potential first step towards resocialization, and previous work supports the use of game and virtual reality media as a novel approach to reach these audiences (see <https://journals.sagepub.com/doi/10.1177/00207640241270748>, [academic.oup.com/bjsw/article-abstract/50/7/2116/5638887](http://academic.oup.com/bjsw/article-abstract/50/7/2116/5638887)). Still, we believe that these forms of media cannot and should not be used by themselves, nor that they should replace traditional approaches such as therapy. The Introduction and Conclusion have been amended to reiterate this point.

With this in mind, real social interaction that does not require the use of games or virtual environments, although ideal and necessary in later stages of the resocialization process, would not fit with the current game-based approaches proposed as first steps, and thus falls outside the scope of the current manuscript.
