# Supplementary material for: Extreme social isolation risk is associated with story-driven, strategic and cooperative-first gameplay preferences
Source: PLOS Ment Health. 2026 Jul 29;3(7):e0000517. doi: 10.1371/journal.pmen.0000517 (PMC13419178; doi:10.1371/journal.pmen.0000517)
Supplement: Attachment — Submitted filename: Response_to_Reviewers_auresp_2.docx [file pmen.0000517.s008.docx]

**For editorial board and reviewers:**

We thank you for your careful consideration of our manuscript and your useful comments. In this letter, we provide point-by-point responses to those comments, addressing questions and problems pointed out by the reviewers. The original reviewer comments will be marked in **bold**, while the responses will be marked in blue. Additions included directly in the manuscript will be marked in red here.

--------------------------------------------------------------------------------------------------------------------

**Reviewer #1:**
**I commend you for the excellent job you did addressing the areas of concern. It is a very interesting, relevant, and potentially impactful manuscript.**

Thank you very much for your feedback.

------------------------------------------------------------------------------------------------------------------------

**Reviewer #3:**

**The Article is a good contribution to the area.**

Thank you very much for your feedback.

------------------------------------------------------------------------------------------------------------------------

**Reviewer #4:**

1) **Line 119: Did participants meet criteria for Internet or gaming addiction? As noted in the Introduction, “games could be a double‑edged sword….” How did the authors assess whether the identified genres are associated with problematic use versus healthy engagement?**

Thank you for your comment. In our current study, we did not assess whether participants met criteria for Internet Gaming Disorder, as our primary focus was to understand the possible relationship between NEET-Hikikomori Risk and gaming preferences. Therefore, our findings do not allow us to determine whether the identified game genres or design elements are associated with healthy or problematic use.

We acknowledge that gaming can function like a “double-edged sword”, as noted in the Introduction. While prior literature *(Stavropoulos, V., Anderson, E. E., Beard, C., Latifi, M. Q., Kuss, D., & Griffiths, M. (2019). A preliminary cross-cultural study of Hikikomori and Internet Gaming Disorder: The moderating effects of game-playing time and living with parents. Addictive Behaviors Reports, 9, 100137.)* suggests that prolonged and immersive gaming may be associated with problematic usage patterns in vulnerable groups such as hikikomori, our study did not directly assess gameplay duration, behavioural addiction, or established IGD criteria.

Furthermore, the “healthiness” of a game feature or genre could be subjective, varying depending on scope, implementation, duration, and other factors. For example, loot boxes have been linked to problem gambling *(Zendle, D., & Cairns, P. (2018). Video game loot boxes are linked to problem gambling: Results of a large-scale survey. PloS one, 13(11), e0206767. ; Zendle, D., & Cairns, P. (2019). Loot boxes are again linked to problem gambling: Results of a replication study. PloS one, 14(3), e0213194. )*.
Despite this, there is evidence to suggest that these associations are exacerbated by factors such as the introduction of real-world spending *(Montiel, I., Basterra-González, A., Machimbarrena, J. M., Ortega-Barón, J., & González-Cabrera, J. (2022). Loot box engagement: A scoping review of primary studies on prevalence and association with problematic gaming and gambling. Plos one, 17(1), e0263177.)*. Kristiansen and Severin point out that a relationship between loot boxes and problem gambling were stronger between those who purchase or sell loot boxes, compared to those who obtain them for free *(Kristiansen S, Severin MC. Loot box engagement and problem gambling among adolescent gamers: Findings from a national survey. Addict Behav. 2020;103: 106254. doi: 10.1016/j.addbeh.2019.106254)*. This highlights that game mechanics are not inherently harmful or beneficial, but may contribute to problematic engagement depending on their design and usage context.

Given the challenges in defining healthy versus unhealthy engagement and game mechanics, the design implications discussed in our Results (e.g., “short play sessions”) should be interpreted as exploratory suggestions for structuring player engagement in this audience. They do not represent validated strategies for promoting healthy behavior, as clarified at the end of our **Discussion** section.

While the proposed design implications (e.g., short play sessions or RPG-like progression) may be relevant for engaging individuals with higher NEET-Hikikomori Risk, it is important to note that the present study does not evaluate whether such engagement is beneficial or detrimental. In the broader literature, distinctions between healthy and problematic gaming are typically based on behavioural outcomes such as loss of control and functional impairment, rather than on specific game mechanics alone. Moreover, operationalizing "healthy'' engagement would require assessing clinical and behavioural indicators beyond the scope of the present study. For example, prior research on randomized reward systems (“loot boxes”) has shown associations with problem gambling severity and gambling-related cognitions (Zendle & Cairns, 2018; Zendle & Cairns, 2019), with evidence suggesting that these relationships may be stronger in contexts involving real-world monetary expenditure (Montiel et al., 2022; Kristiansen & Severin, 2020). Therefore, the implications proposed here should be interpreted as exploratory and hypothesis-generating.

Importantly, this study focuses on identifying engagement patterns within a population at higher risk of social withdrawal, representing a necessary first step in the design of game-based interventions. Without initial engagement, such interventions are unlikely to be adopted or sustained. Therefore, our findings highlight high-isolation risk player preferences and sensitivities, which can inform the development of systems that are both engaging and accessible to this population. Future research should examine how these design elements can be implemented within frameworks that promote healthy and adaptive gaming behaviours.

We also reflected this point in our **limitations**:

Given that our primary focus was the possible relationship between NEET-Hikikomori Risk and gaming preferences, this study did not assess Internet Gaming Disorder, gameplay duration, or other indicators of problematic gaming behaviors. Therefore, while the game design suggestions offered for high-risk players may be relevant in building player engagement for this group, no conclusions can be drawn regarding whether the identified game features promote healthy or unhealthy engagement. Future studies should investigate how specific game design features interact with gameplay duration and behavioural outcomes, to better distinguish between adaptive and maladaptive engagement patterns.

**2) Line 325: Please report the online survey in accordance with the CHERRIES checklist to ensure the quality and transparency of the survey methods and results.**

Thank you for your comment. The CHERRIES list was added as an appendix and its main points were mentioned in the Methodology section, as shown below:

To ensure the quality and facilitate the understanding of our results, the online survey was designed and reported in accordance with the CHERRIES checklist for web-based surveys [62]. An open, voluntary survey was conducted via the Crowdworks platform using a convenience sampling approach, targeting individuals aged 18 years or older residing in Japan with self-reported gaming experience. The questionnaire, implemented in SurveyMonkey, included the validated NEET-Hikikomori Risk (NHR) scale and open-ended questions on gaming preferences. Ethical approval was obtained prior to the study, and informed consent was obtained from all participants prior to participation. Data were collected anonymously, duplicate entries were screened via IP address checks, and incomplete responses were excluded from the final analysis. A detailed CHERRIES checklist [62] is provided in S1_Appendix.

We have also included it at the end of this letter, for easier reading.

**3) Line 334: What was the sampling method? Were participants comparable to the hikikomori profile (e.g., age, gender) reported in representative Japanese samples? If not, weighting should be applied to correct for non‑representativeness and reduce bias.**

Convenience sampling was conducted via Crowdworks because hikikomori and those at high risk of social isolation are traditionally avoidant of social-facing employment and are likely to seek remote, anonymous freelance work. This makes online sampling ideal for our target audience, as it potentially targets a larger population of hikikomori. (For more information on the advantages of convenience sampling, please refer to page 19 of (Landers, R. N., & Behrend, T. S. (2015). An inconvenient truth: Arbitrary distinctions between organizational, Mechanical Turk, and other convenience samples. Industrial and Organizational Psychology, 8(2), 142-164.) )

While recruitment was conducted via convenience sampling, the study maintained high internal validity by utilizing the validated NHR Scale as a psychometric filter to isolate high-risk individuals, ensuring that the sample was representative of multi-generational hikikomori profiles in Japan. This was reflected in the “Participants” sub-section of our Methodology:

To address the potential bias of convenience sampling and ensure the quality of our data, we implemented the validated NEET-Hikikomori Risk (NHR) Scale to precisely identify and isolate individuals at high risk of social isolation. **By applying a predetermined 104-point cut-off score, we were able to distinguish between High-Risk (H-NHR) and Low-Risk (L-NHR) participants, effectively creating a "case-control" structure within our sample**. This selection process resulted in a High-Risk group comprising 52.36% of our participants, confirming that we reached our target demographic. Regarding comparability, our sample (Mean age=38.8; ~50% male) aligns with contemporary Japanese research showing that the hikikomori phenomenon is no longer restricted to male youth but increasingly affects middle-aged individuals across genders(Yong, R. K., Fujita, K., Chau, P. Y., & Sasaki, H. (2020). Characteristics of and gender difference factors of hikikomori among the working-age population: A cross-sectional population study in rural Japan. Nihon Koshu Eisei Zasshi (JAPANESE JOURNAL OF PUBLIC HEALTH), 67(4), 237-246.).

Because our research goals focus on comparing the specific gaming preferences and psychological needs of these two psychometrically-defined groups rather than estimating nationwide prevalence, the use of the validated NHR scale provides the necessary internal validity for our comparative analysis without the requirement for population-wide weighting.

**4) Line 364: Please report the psychometric properties of the NHR measure used in this study (e.g., reliability, validity).**

The reliability and validity of the NEET-Hikikomori Scale can be found in its original article*(Uchida, Y., & Norasakkunkit, V. (2015). The NEET and Hikikomori spectrum: Assessing the risks and consequences of becoming culturally marginalized. Frontiers in psychology, 6, 1117.)*.
We have adjusted our manuscript to reflect this information, as written below.

According to Norasakkunkit & Uchida, a reliability analysis was done and showed that the scale's combined items could represent an overall NEET-Hikikomori Risk, indicated by a Cronbach's alpha of 0.82. Study 2, present in the same paper, also investigated and confirmed the validity of the scale, finding that NHR scores correspond with varying degrees of marginalized strata in a nationwide Japanese sample.

**5) Line 410: Why are analytic procedures described in the Results section? The survey results appear to be missing from that section.**

Thank you for your comment. The analytic procedures described in the “Data treatment” subsections were moved to the “Survey Analysis” part within the “Methodology” section.

The survey results, as well as their interpretation, can be found in the renamed “Genre Analysis -- Quantitative results” and “Game Preferences Analysis - Qualitative results” sections.

**6) Line 440: How many responses were marked “Not Available,” and what is the potential impact on the findings?**

A: A total of 1029 individually mentioned games were found. From the games not found, seven (0.68% of total mentions) were game types (like "dress-up") or board/table games (e.g., "Air Hockey"). Since these represented generic game types, or games that are not necessarily video games (such as board games), they did not fit with our scope of investigating video-game genres, and thus were excluded from the analysis.

Furthermore, 69 individual games were not found in our database search, which accounted for 113 (10.98%) of the total valid mentions from players. From these mentions, most were either too new at the time, or too niche to the Japanese market to be found in our chosen gaming database. Nevertheless, the remaining ~89% of mentions (916, with ~51.9% of them coming from high-risk participants, maintaining the case-control structure, proper for the intended analysis) still provide an adequate sample size, and our results align with previous hikikomori gaming literature (Kubo et al. Hikikomori and gaming disorder tendency: A case-control online survey for non-working adults, 2023).

The manuscript was adjusted to include this information:

In total, 432 distinct game titles were collected from RAWG API, in a total of 1029 mentions. These yielded 1377 genre entries, composed of a pair of "Participant ID" and "genre mentioned", and 5943 keyword entries. Participants also mentioned 69 distinct games, across 113 total mentions (10.98% of our total number of mentions pre-exclusions) that could not be found in the RAWG API. These were marked as "Not Available'' and removed from the dataset. Many of these entries were either too niche to the Japanese market to be found in our chosen database, or too new to have detailed entries yet. Nevertheless, the remaining mentions still provide an adequate sample for this study.

**7) Line 515: Did you implement peer debriefing and member checking to ensure the trustworthiness of the qualitative themes?**

Yes, peer debriefing was implemented via triangulation through three coders with different backgrounds (psychology and engineering). We utilized "code standardization" and "final coder agreement" processes, requiring consensus from at least two of three coders) to minimize individual bias and ensure trustworthiness.

While member checking was not possible due to participant anonymity and an online data collection process, our internal verification steps of "code standardization" and "final coder agreement", described in the manuscript (Methodology section, “Survey Analysis” sub-section), served as the primary method to ensure the credibility of our data and results.

**8) Line 798: The genre categories (Role‑playing, Puzzle, Action, Simulation, Adventure) are very broad. How do these findings translate into practical implications?**

While the genre categories are broad, they serve as a foundation for proposing genre-independent design suggestions that can be implemented in several of these categories. For instance, "RPG-like progression" mechanics, described in more detail in our manuscript, can be found in Role-Playing games (as seen in classical Final Fantasy titles) and Action games (such as the action RPG Deus Ex series). Similarly, "Short play session" can be applied to several, if not all, of the previously mentioned genre categories.

In short, the genre categories are used to guide and complement the suggestions of more specific game design features that were described in our qualitative results and grounded in the Self-Determination Theory (SDT) (as shown in Table 3). For example, to translate the broad “Action” genre into a practical intervention, our findings suggest implementing “RPG-like progression” and “Simple mechanics”, which could fulfill Competence needs. Furthermore, for Relatedness needs, we recommend shifting the social focus of these genres from competition to cooperative gameplay, to mitigate the reported fear of being a nuisance and of “toxic interactions”.

This was described in more detail in the “Recommendations for design” sub-section:

While the identified genres (Action, RPG, etc.) provide a broad overview of the gaming landscape favoured by hikikomori players and corroborated by past research, the practical utility of these findings lies in the identification of categories that can be broken down further into specific characteristics of each genre or broader interchangeable features useable in any of them. This can then be translated to needs-based design requirements grounded on the SDT.

We propose a layered design approach, where these broad game genres serve both as the foundational "vessels'' for interventions, and as a basis for future studies that can investigate particular characteristics of each genre. This foundation must then be refined by mapping each genre's inherent strengths and characteristics to the intrinsic needs of the SDT (Autonomy, Competence, Relatedness), while applying the specific functional and non-functional requirements identified in our qualitative analysis (see Table 2).

For example, a broad "Action'' or "RPG'' vessel is practically translated into an effective intervention for the H-NHR group by implementing "RPG-like progression'' and "simple mechanics'', to fulfill Competence needs. This specific implementation ensures that players experience a sense of growth without the high-difficulty hurdles or negative feedback that typically lead to disengagement in high-risk individuals. Similarly, "short play sessions'' can be implemented across all preferred genres to fulfill Autonomy by allowing players to manage physical and visual fatigue at their own pace. Furthermore, social mechanics within these genres should be steered toward "cooperative gameplay'' to meet Relatedness needs, while bypassing the social anxiety and toxic interactions reported by this demographic.

By layering these specific, qualitative requirements onto familiar genres, developers can create game-based interventions that are both engaging and accessible to a population sensitive to social rejection and high difficulty.

**9) Which genres were less popular among individuals with hikikomori, and why?**

According to our Appendix results (Table 6. Genre Analysis - Mentions per NHR group), we can see that the three types of genre categories less mentioned by those with higher risk of social withdrawal are "Family", "Sports", and lastly, "Fighting".

Although we cannot say with certainty why high-risk players seem to be less interested in these categories, one possible reason for the low mention of "Family"-related games is that those with a high risk of social isolation might already live by themselves, and therefore do not have family members nearby with whom to share such kinds of games.

As for low mentions of the "Fighting" category, it might be related to how high-risk hikikomori people react to stressful situations. This is corroborated by previous studies showing that individuals with higher hikikomori risk were less likely to persist on challenging tasks when faced with negative feedback (Norasakkunkit and Uchida. Psychological consequences of postindustrial anomie on self and motivation among Japanese youth, 2011), and thus the emotional cost of losing in competitive fighting games may steer this audience away from this genre.

**CHERRIES Checklist:**

**1) Design**

**Target Population:** People with gaming experience who self-report interest in video games, aged 18+, born, raised, and currently residing in Japan

**Survey Type:** An open survey (accessible via the Crowdworks platform to any user meeting the criteria) with a convenience sample.

**Technical Platform:** Implemented using the SurveyMonkey platform.

**2) IRB Approval**

**IRB Approval:** Approved by the Institutional Review Board of the Nara Institute of Science and Technology (Review Number: 2022-I-25).

Informed Consent: Detailed written instructions were provided prior to the start of the survey; participants gave informed written consent by choosing to participate.

**Data Protection**: The survey was anonymous. IP addresses were collected automatically by the SurveyMonkey platform but were not used or disclosed during any part of the study.

**3) Development and Pre-testing**

**Development:** The survey used the validated NEET-Hikikomori Risk (NHR) Scale and customized open questions about participants’ interests on certain types of games.

**Pre-testing/Pilot:** The survey was manually pilot-tested by the lead author before full deployment.

**4) Recruitment Process and Sample Description**

**Open survey versus closed survey:** Open survey through Crowdworks (a Japanese crowdsourcing/freelance website).

**Contact mode:** Internet, conducted in two rounds during February 2023.

.

**Advertising the survey:** Advertised through the Crowdworks platform.

**5) Survey Administration**

**Web/email:** Web

**Context:** Crowdworks is Japan’s largest online crowdsourcing platform, connecting companies with freelancers and individuals for various work tasks. For our work, it was used to find participants for a survey.

**Mandatory/voluntary:** Voluntary

**Incentives:** Monetary reward of 500JPY upon completion of the questionnaire.

**Time/date:** The survey was conducted in two rounds during February 2023.

**Randomization of items or questionnaires:** The order of the questionnaire items was not randomized.

**Adaptive questioning:** The descriptive questions asking about participants’ specific favorite games and thoughts on single/multiplayer games was only presented when participants answered “Yes” to the first question of the questionnaire (“Do you play games?”)

**Number of Items:** The questionnaire contained between 40 and 50 items, depending on the participant's answers.

**Number of screens (pages):** Not recorded

**Completeness check:** Completeness check was done on all answers using R, and participants who did not answer all mandatory questions were excluded from the final analysis.

**Review step:** Participants can go back to modify answers while answering the survey, before they submit their final answer.

**6) Response rates**

**Unique site visitor:** Crowdworks collect IP addresses of participants as part of its metadata. This was used to check for possible duplicate participants, but no duplicates were found.

**View rate (Ratio of unique survey visitors/unique site visitors)**	N/A

**Participation rate (Ratio of unique visitors who agreed to participate/unique first survey page visitors):** N/A

**Completion rate (Ratio of users who finished the survey/users who agreed to participate):** The total number of collected responses from users who agreed to participate was 676. The number of people who did not complete the survey to the end was 72.

Completion rate: 1 - 72/676 = 89.3%

**7) Preventing multiple entries from the same individual**

**Cookies used:** N/A

**IP check:** Yes, IP address was collected as meta-data, and if any duplicate addresses were found, only the first one would be considered. No duplicate entries were found.

**Log file analysis:** N/A

**Registration:** N/A

**8) Analysis**

**Handling of incomplete questionnaires:** Incomplete questionnaires were removed entirely from the analysis.

**Questionnaires submitted with an atypical timestamp:** N/A

**Statistical correction:** Not used
