# Supplementary material for: Extreme social isolation risk is associated with story-driven, strategic and cooperative-first gameplay preferences
Source: PLOS Ment Health. 2026 Jul 29;3(7):e0000517. doi: 10.1371/journal.pmen.0000517 (PMC13419178; doi:10.1371/journal.pmen.0000517)
Supplement: Peer review history

# Peer review: Decision Letter 0

**DOI:** 10.1371/journal.pmen.0000517.r001

**Authors:** David Onchonga

16 Jul 2025

PMEN-D-25-00246

Extreme social isolation risk is associated with story-driven, strategic and cooperative-first gameplay preferences

PLOS Mental Health

Dear Dr. Confessor,

Thank you for submitting your manuscript to PLOS Mental Health. After careful consideration, we feel that it has merit but does not fully meet PLOS Mental Health’s publication criteria as it currently stands. Therefore, we invite you to submit a revised version of the manuscript that addresses the points raised during the review process.

Please submit your revised manuscript by Aug 30 2025 11:59PM. If you will need more time than this to complete your revisions, please reply to this message or contact the journal office at mentalhealth@plos.org. When you're ready to submit your revision, log on to https://www.editorialmanager.com/pmen/ and select the 'Submissions Needing Revision' folder to locate your manuscript file.

Please include the following items when submitting your revised manuscript:

- A rebuttal letter that responds to each point raised by the editor and reviewer(s). You should upload this letter as a separate file labeled 'Response to Reviewers'.
- A marked-up copy of your manuscript that highlights changes made to the original version. You should upload this as a separate file labeled 'Revised Manuscript with Track Changes'.
- An unmarked version of your revised paper without tracked changes. You should upload this as a separate file labeled 'Manuscript'.

Guidelines for resubmitting your figure files are available below the reviewer comments at the end of this letter.

We look forward to receiving your revised manuscript.

Kind regards,

David Onchonga, Ph.D.

Academic Editor

PLOS Mental Health

Journal Requirements:

1. We ask that a manuscript source file is provided at Revision. Please upload your manuscript file as a .doc, .docx, .rtf or .tex.

2. Thank you for uploading your study's underlying data set. Unfortunately, the repository you have noted in your Data Availability statement does not qualify as an acceptable data repository according to PLOS's standards.

At this time, please upload the minimal data set necessary to replicate your study's findings to a stable, public repository (such as figshare or Dryad) and provide us with the relevant URLs, DOIs, or accession numbers that may be used to access these data. For a list of recommended repositories and additional information on PLOS standards for data deposition, please see https://journals.plos.org/plosone/s/recommended-repositories.

3. If the reviewer comments include a recommendation to cite specific previously published works, please review and evaluate these publications to determine whether they are relevant and should be cited. There is no requirement to cite these works unless the editor has indicated otherwise.

Additional Editor Comments (if provided):

[Note: HTML markup is below. Please do not edit.]

Reviewers' comments:

Reviewer's Responses to Questions

**Comments to the Author**

1. Does this manuscript meet PLOS Mental Health’s publication criteria? Is the manuscript technically sound, and do the data support the conclusions? The manuscript must describe methodologically and ethically rigorous research with conclusions that are appropriately drawn based on the data presented.

Reviewer #1: Yes

Reviewer #2: Partly

**********

2. Has the statistical analysis been performed appropriately and rigorously?

Reviewer #1: Yes

Reviewer #2: Yes

**********

3. Have the authors made all data underlying the findings in their manuscript fully available (please refer to the Data Availability Statement at the start of the manuscript PDF file)?

The PLOS Data policy requires authors to make all data underlying the findings described in their manuscript fully available without restriction, with rare exception. The data should be provided as part of the manuscript or its supporting information, or deposited to a public repository. For example, in addition to summary statistics, the data points behind means, medians and variance measures should be available. If there are restrictions on publicly sharing data—e.g. participant privacy or use of data from a third party—those must be specified.

Reviewer #1: Yes

Reviewer #2: Yes

**********

4. Is the manuscript presented in an intelligible fashion and written in standard English?

PLOS Mental Health does not copyedit accepted manuscripts, so the language in submitted articles must be clear, correct, and unambiguous. Any typographical or grammatical errors should be corrected at revision, so please note any specific errors here.

Reviewer #1: Yes

Reviewer #2: Yes

**********

5. Review Comments to the Author

Please use the space provided to explain your answers to the questions above. You may also include additional comments for the author, including concerns about dual publication, research ethics, or publication ethics. (Please upload your review as an attachment if it exceeds 20,000 characters)

Reviewer #1: I appreciated the opportunity to review your article. I thought that your writing was well reasoned and easy to follow. I have some fundamental concerns about seeing a gaming intervention meeting the criteria laid out for SDT. I understand and don't necessarily disagree that there could be short-term and limited benefit in the SDT categories of autonomy, competence, and relatedness. However, my concern is that the article seems to suggest that this would be an overall viable intervention approach, but I don't see how this translates to something concrete in the real world. I don't see the leap from potentially improved online interaction and isolated improved mood to a functional social being. I think that using gaming as a potential therapeutic response drives us further into a dystopian realm where reality based human interaction becomes increasingly unlikely. I do think your research should be published, however, in my opinion I think some commentary on whether this could ever empower a person to successfully improve their mental health outside of the virtual context would be appropriate.

Reviewer #2: I enjoyed reading your work, though do consider some overarching features need revision / expansion (see uploaded response to your your paper). You might also consider whether there is a conflict of interest (being game designers yourselves). There is a need for some coverage of neurodiversity, that you hint at, and beyond this, you might consider that masking gaming more suitable for those who exhibit H-NHR may be critiqued too. The real world might not be so accommodating, and so some consideration of how gaming might for example encourage some robustness or resilience as a narrative seems required, to demonstrate a purpose, otherwise the work might be critiqued for encouraging further entrenchment to isolation in H-NHR people. Some sequencing in the paper could be revised, and a statement on positionality is also required. There also seems to be an error in reporting the chi square in the general discussion, and an appreciation of the mental health implications seems warranted here too. some clarity on the use of self determination theory as you progress through your paper would be good also, as might expansions on behavioural and cognitive components you mention. clearer link to mental health literature with regards to your introduction, thematic analysis and recommendations seems warranted too. A fuller consideration of H-NHR and gaming disorder seems apt, and a consideration that gaming might be a component to be managed, so that real social interaction might be encouraged also, for greater wellbeing. The omission of such arguments may prevent a balanced appraisal of the needs for those wo exhibit Hikikomori.

Your work is intriguing, though honing it for a clinical audience would benefit your wish to publish it here.

**********

6. PLOS authors have the option to publish the peer review history of their article (what does this mean?). If published, this will include your full peer review and any attached files.

**Do you want your identity to be public for this peer review?**  If you choose “no”, your identity will remain anonymous but your review may still be made public.

For information about this choice, including consent withdrawal, please see our Privacy Policy.

Reviewer #1: **Yes:**  Noah Hansen

Reviewer #2: No

**********

[NOTE: If reviewer comments were submitted as an attachment file, they will be attached to this email and accessible via the submission site. Please log into your account, locate the manuscript record, and check for the action link “View Attachments”. If this link does not appear, there are no attachment files.]

While revising your submission, please upload your figure files to the Preflight Analysis and Conversion Engine (PACE) digital diagnostic tool, https://pacev2.apexcovantage.com/. PACE helps ensure that figures meet PLOS requirements. To use PACE, you must first register as a user. Registration is free. Then, login and navigate to the UPLOAD tab, where you will find detailed instructions on how to use the tool. If you encounter any issues or have any questions when using PACE, please email PLOS at figures@plos.org. Please note that Supporting Information files do not need this step.

# Author comment: Author response to Decision Letter 1

**DOI:** 10.1371/journal.pmen.0000517.r002

31 Aug 2025

Attachment Submitted filename: Response to Reviewers.docx

# Peer review: Decision Letter 1

**DOI:** 10.1371/journal.pmen.0000517.r003

**Authors:** Steve Zimmerman

15 Feb 2026

PMEN-D-25-00246R1

Extreme social isolation risk is associated with story-driven, strategic and cooperative-first gameplay preferences

PLOS Mental Health

Dear Dr. Confessor,

Thank you for submitting your manuscript to PLOS Mental Health. After careful consideration, we feel that it has merit but does not fully meet PLOS Mental Health’s publication criteria as it currently stands. Therefore, we invite you to submit a revised version of the manuscript that addresses the points raised during the review process.

The manuscript has been evaluated by three reviewers, and their comments are available below.

Could you please revise the manuscript to carefully address the concerns raised?

Please submit your revised manuscript by Mar 30 2026 11:59PM. If you will need more time than this to complete your revisions, please reply to this message or contact the journal office at mentalhealth@plos.org. When you're ready to submit your revision, log on to https://www.editorialmanager.com/pmen/ and select the 'Submissions Needing Revision' folder to locate your manuscript file.

Please include the following items when submitting your revised manuscript:

- A letter that responds to each point raised by the editor and reviewer(s). You should upload this letter as a separate file labeled 'Response to Reviewers'.
- A marked-up copy of your manuscript that highlights changes made to the original version. You should upload this as a separate file labeled 'Revised Manuscript with Track Changes'.
- An unmarked version of your revised paper without tracked changes. You should upload this as a separate file labeled 'Manuscript'.

Guidelines for resubmitting your figure files are available below the reviewer comments at the end of this letter.

We look forward to receiving your revised manuscript.

Kind regards,

Steve Zimmerman, PhD

PLOS Staff Editor

Journal Requirements:

If the reviewer comments include a recommendation to cite specific previously published works, please review and evaluate these publications to determine whether they are relevant and should be cited. There is no requirement to cite these works unless the editor has indicated otherwise.

Additional Editor Comments (if provided):

[Note: HTML markup is below. Please do not edit.]

Reviewers' comments:

Reviewer's Responses to Questions

**Comments to the Author**

1. If the authors have adequately addressed your comments raised in a previous round of review and you feel that this manuscript is now acceptable for publication, you may indicate that here to bypass the “Comments to the Author” section, enter your conflict of interest statement in the “Confidential to Editor” section, and submit your “Accept” recommendation.

Reviewer #1: All comments have been addressed

Reviewer #3: All comments have been addressed

Reviewer #4: (No Response)

**********

2. Does this manuscript meet PLOS Mental Health’s publication criteria? Is the manuscript technically sound, and do the data support the conclusions? The manuscript must describe methodologically and ethically rigorous research with conclusions that are appropriately drawn based on the data presented.

Reviewer #1: Yes

Reviewer #3: Yes

Reviewer #4: (No Response)

**********

3. Has the statistical analysis been performed appropriately and rigorously?

Reviewer #1: Yes

Reviewer #3: Yes

Reviewer #4: (No Response)

**********

4. Have the authors made all data underlying the findings in their manuscript fully available (please refer to the Data Availability Statement at the start of the manuscript PDF file)?

The PLOS Data policy requires authors to make all data underlying the findings described in their manuscript fully available without restriction, with rare exception. The data should be provided as part of the manuscript or its supporting information, or deposited to a public repository. For example, in addition to summary statistics, the data points behind means, medians and variance measures should be available. If there are restrictions on publicly sharing data—e.g. participant privacy or use of data from a third party—those must be specified.

Reviewer #1: Yes

Reviewer #3: No

Reviewer #4: (No Response)

**********

5. Is the manuscript presented in an intelligible fashion and written in standard English?

PLOS Mental Health does not copyedit accepted manuscripts, so the language in submitted articles must be clear, correct, and unambiguous. Any typographical or grammatical errors should be corrected at revision, so please note any specific errors here.

Reviewer #1: Yes

Reviewer #3: Yes

Reviewer #4: (No Response)

**********

6. Review Comments to the Author

Please use the space provided to explain your answers to the questions above. You may also include additional comments for the author, including concerns about dual publication, research ethics, or publication ethics. (Please upload your review as an attachment if it exceeds 20,000 characters)

Reviewer #1: I commend you for the excellent job you did addressing the areas of concern. It is a very interesting, relevant, and potentially impactful manuscript.

Reviewer #3: The Article is a good contribution to the area.

Reviewer #4: The study examines the relationship between extreme social isolation risk and gameplay preferences. While the research objectives are important and with practical implications that games may facilitate engagement and support mental health among hikikomori, there are major methodological concerns. My comments are as follows:

Line 119: Did participants meet criteria for Internet or gaming addiction? As noted in the Introduction, “games could be a double‑edged sword….” How did the authors assess whether the identified genres are associated with problematic use versus healthy engagement?

Line 325: Please report the online survey in accordance with the CHERRIES checklist to ensure the quality and transparency of the survey methods and results.

Line 334: What was the sampling method? Were participants comparable to the hikikomori profile (e.g., age, gender) reported in representative Japanese samples? If not, weighting should be applied to correct for non‑representativeness and reduce bias.

Line 364: Please report the psychometric properties of the NHR measure used in this study (e.g., reliability, validity).

Line 410: Why are analytic procedures described in the Results section? The survey results appear to be missing from that section.

Line 440: How many responses were marked “Not Available,” and what is the potential impact on the findings?

Line 515: Did you implement peer debriefing and member checking to ensure the trustworthiness of the qualitative themes?

Line 798: The genre categories (Role‑playing, Puzzle, Action, Simulation, Adventure) are very broad. How do these findings translate into practical implications? Which genres were less popular among individuals with hikikomori, and why?

**********

7. PLOS authors have the option to publish the peer review history of their article (what does this mean?). If published, this will include your full peer review and any attached files.

**Do you want your identity to be public for this peer review?**  If you choose “no”, your identity will remain anonymous but your review may still be made public.

For information about this choice, including consent withdrawal, please see our Privacy Policy.

Reviewer #1: No

Reviewer #3: No

Reviewer #4: No

**********

[NOTE: If reviewer comments were submitted as an attachment file, they will be attached to this email and accessible via the submission site. Please log into your account, locate the manuscript record, and check for the action link “View Attachments”. If this link does not appear, there are no attachment files.]

-->Figure Resubmissions:

While revising your submission, we strongly recommend that you use PLOS’s NAAS tool (https://ngplosjournals.pagemajik.ai/artanalysis) to test your figure files. NAAS can convert your figure files to the TIFF file type and meet basic requirements (such as print size, resolution), or provide you with a report on issues that do not meet our requirements and that NAAS cannot fix.

After uploading your figures to PLOS’s NAAS tool - https://ngplosjournals.pagemajik.ai/artanalysis, NAAS will process the files provided and display the results in the “Uploaded Files” section of the page as the processing is complete. If the uploaded figures meet our requirements (or NAAS is able to fix the files to meet our requirements), the figure will be marked as “fixed” above. If NAAS is unable to fix the files, a red “failed” label will appear above. When NAAS has confirmed that the figure files meet our requirements, please download the file via the download option, and include these NAAS processed figure files when submitting your revised manuscript.

# Author comment: Author response to Decision Letter 2

**DOI:** 10.1371/journal.pmen.0000517.r004

28 Apr 2026

Attachment Submitted filename: Response_to_Reviewers_auresp_2.docx

# Editor report: Decision Letter 2

**DOI:** 10.1371/journal.pmen.0000517.r005

**Authors:** Lucas Murrins Marques

21 May 2026

Extreme social isolation risk is associated with story-driven, strategic and cooperative-first gameplay preferences

PMEN-D-25-00246R2

Dear Mr. Confessor,

We are pleased to inform you that your manuscript 'Extreme social isolation risk is associated with story-driven, strategic and cooperative-first gameplay preferences' has been provisionally accepted for publication in PLOS Mental Health.

Before your manuscript can be formally accepted you will need to complete some formatting changes, which you will receive in a follow up email. A member of our team will be in touch with a set of requests.

Please note that your manuscript will not be scheduled for publication until you have made the required changes, so a swift response is appreciated.

IMPORTANT: The editorial review process is now complete. PLOS will only permit corrections to spelling, formatting or significant scientific errors from this point onwards. Requests for major changes, or any which affect the scientific understanding of your work, will cause delays to the publication date of your manuscript.

If your institution or institutions have a press office, please notify them about your upcoming paper to help maximize its impact. If they'll be preparing press materials, please inform our press team as soon as possible -- no later than 48 hours after receiving the formal acceptance. Your manuscript will remain under strict press embargo until 2 pm Eastern Time on the date of publication. For more information, please contact mentalhealth@plos.org.

Thank you again for supporting Open Access publishing; we are looking forward to publishing your work in PLOS Mental Health.

Best regards,

Lucas Murrins Marques

Academic Editor

PLOS Mental Health

***********************************************************

Reviewer Comments (if any, and for reference):
